# Mineral reaction kinetics constrain the length scale of rock matrix diffusion

**DOI:** 10.1038/s41598-020-65113-x

**Published:** 2020-05-18

**Authors:** R. A. Wogelius, A. E. Milodowski, L. P. Field, R. Metcalfe, T. Lowe, A. van Veelen, G. Carpenter, S. Norris, B. Yardley

**Affiliations:** 1 University of Manchester, School of Earth and Environmental Sciences, Williamson Research Centre for Molecular Environmental Science & Interdisciplinary Centre for Ancient Life, Manchester, M13 9PL UK; 2 British Geological Survey, Environmental Science Centre, Nicker Hill, Keyworth, Nottingham, NG12 5GG UK; 3 Quintessa Limited, First Floor, West Wing, Videcom House, Newtown Road, Henley-on-Thames, Oxfordshire, RG9 1HG UK; 4 Henry Moseley X-ray Imaging Facility, The University of Manchester, Upper Brook Street, Manchester, M13 9PY UK; 5 Material Science and Technology Division, Los Alamos National Laboratory, Los Alamos, NM 87545 USA; 6 NSG Environmental Ltd, Festival House, Jessop Avenue, Cheltenham, Gloucestershire GL50 3SH UK; 7 Radioactive Waste Management Limited, Building 587, Curie Avenue, Harwell Science and Innovation Campus, Didcot, Oxfordshire OX11 0RH UK; 8 School of Earth and Environment, University of Leeds, Leeds, LS2 9JT UK

**Keywords:** Environmental sciences, Hydrology

## Abstract

Mass transport by aqueous fluids is a dynamic process in shallow crustal systems, redistributing nutrients as well as contaminants. Rock matrix diffusion into fractures (void space) within crystalline rock has been postulated to play an important role in the transient storage of solutes. The reacted volume of host rock involved, however, will be controlled by fluid-rock reactions. Here we present the results of a study which focusses on defining the length scale over which rock matrix diffusion operates within crystalline rock over timescales that are relevant to safety assessment of radioactive and other long-lived wastes. Through detailed chemical and structural analysis of natural specimens sampled at depth from an active system (Toki Granite, Japan), we show that, contrary to commonly proposed models, the length scale of rock matrix diffusion may be extremely small, on the order of centimetres, even over timescales of millions of years. This implies that in many cases the importance of rock matrix diffusion will be minimal. Additional analyses of a contrasting crystalline rock system (Carnmenellis Granite, UK) corroborate these results.

## Introduction

Models of mass transport in fractured crystalline lithologies typically assumed that rock matrix diffusion (RMD) into the matrix led to lateral fluid flux, with diffusion perpendicular to the flow path potentially occurring on the meter to tens of meters length scale^1^. Conceptual diagrams in hydrogeology textbooks emphasized that the length scale of diffusion perpendicular to the fracture surface would be large compared to fracture aperture width (e.g. Figure 2.40^2^). This diffusive process may attenuate solute concentrations, cause lag in breakthrough curves, and extend release times long after a chemical pulse has ended at the source (a phenomenon known as “transient storage”). Because transient storage reduces the aqueous concentrations of contaminants in the early stages of release from a source, contaminant transport models that include RMD will generally indicate lower environmental risks than models without RMD. However, transient storage also implies that risks will persist for longer time periods if RMD has been a significant process. Therefore, RMD needs to be understood in order to successfully predict chemical transport in the subsurface. Field and laboratory experiments have shown that losses of trace components from flowing groundwater are often greater than predicted by simple surface adsorption, but the length scales over which RMD operates are not well-constrained. Recent field tracer tests have shown however that rock matrix diffusion in short duration experiments is not the best explanation for observed breakthrough tailing^3–6^. Indeed, it has been concluded^3^ that discontinuous advection, rather that RMD, provides a better theoretical model for tailing in hydrological field experiments run over relatively short timescales. However tracer tests are typically run over time periods of days to weeks, and matrix diffusion is not a time reversible process^5^. Therefore when considering fluid flux through a geological fracture system that may persist over time scales in excess of hundreds or thousands of years, it is yet unclear whether RMD may significantly impact solute transport processes. Critically important is that models of RMD typically give little consideration to processes besides diffusion and adsorption that may affect fluid chemistry, such as mineral precipitation and dissolution.

For these reasons RMD is an important process to constrain with respect to predicting chemical transport in the subsurface. Models typically assume that RMD will act to include a large volume of the lithology.

Previous research has also indicated that length-scales of RMD are likely to be site specific, for example studies of the Japanese Kurihashi Granodiorite, Tono Granite (Japan) and Grimsel Granodiorite (Switzerland) postulate length scales of only 10 to 100 mm^7–12^. Studies of low-temperature wallrock alteration from > 800 fractures in volcanic tuff from the Borrowdale Volcanic Group (BVG, UK) show length scales of <1 to 15 mm with mineralogical alteration affecting only 0.4% of the rock mass^13^, although perturbation of some trace elements and U-series disequilibrium was detected to at least 30 mm from the fractures^13,14^. The U-series disequilibrium studies demonstrated wallrock enrichment of U had occurred over these length scales within the past 1 Ma^14^. Research concerning underground CO_2_ storage finds CO_2_ diffusion to be extremely slow into caprock, with cm to metre penetration taking 10,000 to 100,000 years^15,16^. Furthermore, experimental work in the hydrothermal P-T range has shown that mineral precipitation can cause an exponential decrease in permeability of granite^17^. In the Hot Fractured Rock project in the Soultz-sous-Forêts region in France, it was also seen that granite permeability may significantly decrease as a function of secondary mineral precipitation^18^. Thus, there is a documented discrepancy between the originally postulated theoretical length scale of RMD and the much shorter effective length scales seen in short term experiments and many natural systems.

The work presented here seeks to directly determine how alteration processes impact mass transport in natural fracture systems. Our key hypothesis is: ‘Direct observations of specimens that have been reacted with aqueous fluids at depth over geological time periods will provide measurements that will improve our understanding of the characteristic length of rock matrix diffusion in crystalline rock’ Our auxiliary question is: ‘What chemical and physical processes can be directly observed which will assist in defining this length scale?’ Our key findings will be illustrated by focussing on two well-constrained rock cores sampled from an active groundwater system in the Toki Granite (central Japan). These cores represent two points on a continuum of reaction progress from nearly pristine to significantly altered. We will highlight the general applicability of our findings by comparing the Toki Granite results to another crystalline lithology, the Carnmenellis Granite (Cornwall, UK).

## Results

### Toki Granite model system description

The Toki Granite pluton (~70 Mya) is one of several Cretaceous granitic bodies in central Japan^19,20^. The intrusion consists of marginal muscovite–biotite granite, which grades through hornblende–biotite granite to biotite granite in central parts^19,20^. Plagioclase compositions range from oligoclase to albite (Ab_71–94_) while K-feldspar is microcline (Or_90-97_) [electron microprobe analyses, see Supplementary Table 1]. Chlorite, illite, and epidote are common secondary phases^19^.

The studied specimens came from the MIU-3 borehole which was drilled by the Japan Nuclear Cycle Development Institute (JNC) near the town of Mizunami in Gifu Prefecture, central Honshu^21–27^ (see also Supplementary Note 1). Groundwater in the borehole actively flows through a fracture network and is of meteoric origin with the recharge zone located approximately 6 km to the northeast. In and around the MIU-3 borehole, all the encountered groundwater is of the Na-HCO_3_ type, with salinity up to c. 200 mg/L^28^. Water analyses from closest to the MIU-3/8 and MIU-3/10 rock samples are shown in Supplementary Table 2. ^14^C data suggests that groundwaters in the middle part of the sedimentary rock sequence near the MIU borehole have resided there for ~9.3 kya, while the groundwater residence in the deeper granite is estimated to be>50 ka^25,28^.

U-series isotope data for sub-samples of Toki Granite taken from borehole MIZ-1 (~2.5 km to the southeast of the MIU-3 borehole) have recently been reported^29^. The U-series isotope data came from sub-samples of rock cores at approximately 190 m depth and 270 m depth below the ground surface. In the case of the 190 m deep core, 11 sub-samples along a 70 mm long profile perpendicular to a fracture were analysed. For the core 270 m deep, 11 additional sub-samples were taken from each of two 70 mm long profiles, one on each side of a fracture, and perpendicular to it. The ^234^U/^238^U and ^230^Th/^238^U ratios are approximately 1 along two of the three profiles (i.e. the samples from c. 190 m depth and one of the two sample profiles from c. 270 m depth). In contrast, in both the shallower and deeper core samples, the ^226^Ra/^230^Th ratio is significantly higher closer to the fracture. These results are consistent with U and Th having experienced minimal mass transfer, but with ^226^Ra having been added to the rock via mineral-fluid interaction.

The two samples studied in detail were specifically chosen as examples of wallrock adjacent to fractures containing the most recent mineralisation and potentially associated with present groundwater flow paths (see Supplementary Note 1 for further details of sample selection criteria). They come from borehole MIU-3; sample MIU-3/8 was obtained from 555 m depth, and MIU-3/10 is from 522 m depth, and have therefore experienced nearly identical ambient conditions. These will be compared with another crystalline rock (Carnmenellis Granite (310 Mya, 2280 m depth, Rosemanowes, Cornwall), similar to the Toki Granite but with less indication of active fluid flow or mineralogical alteration.

### Void space analysis

**Figure 1:**
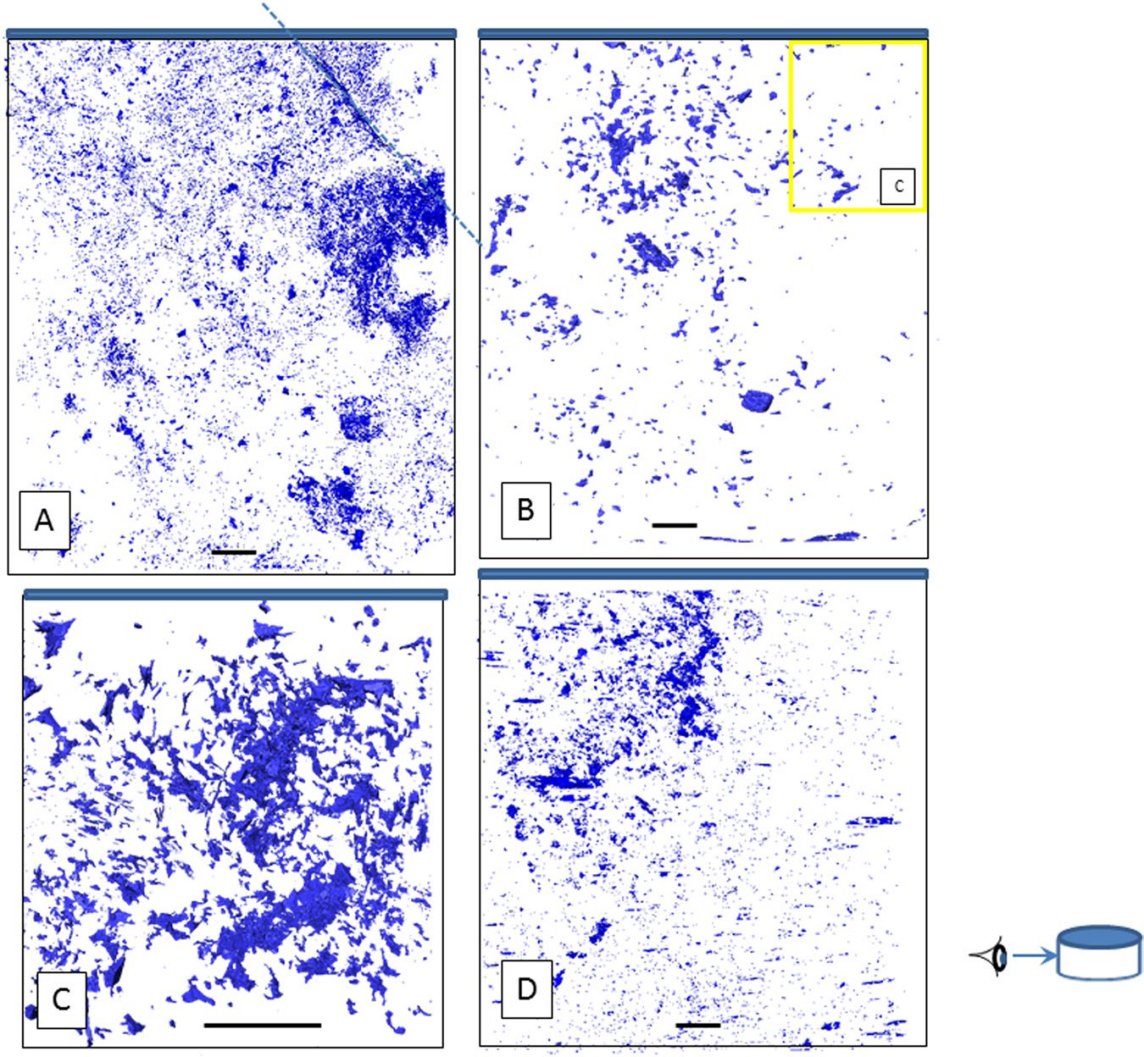
Pore sizes and pore distributions within the Toki and Carnmenellis granite proximate to primary fractures (indicated by solid blue regions at upper surfaces) as determined via X–ray CT analysis. (**A**) In the less reacted Toki MIU-3/8 (11 mm diameter core, 15 μm resolution), pores with a minimum dimension of 100 μm occupy 0.11% of the volume, and are distributed as a “cloud” with only one potential connected parasitic fracture indicating limited connectivity (dashed line). (**B**) For the more reacted Toki MIU-3/10 (11 mm diameter core, 15 μm resolution), pore volume is significantly reduced by nearly a factor of two (0.06% volume) and there is minimal connectivity. Yellow box indicates the relative sizes of scans B and C. (**C**) A higher resolution scan of MIU-3/10 (5 mm diameter core, 7 μm resolution) showing pores with a minimum dimension of 50 μm, indicates that when smaller pores are included the pore volume increases slightly as would be expected (0.84%) but even within 3 mm of the fracture the connectivity and pore volumes are minimal. (**D**) Carmenellis granite scan (11 mm diameter core, 15 μm resolution) also showing little connectivity between pores and a pore volume similar to MIU-3/10 (0.05%). (Scale bars = 1 mm. Inset shows view perspective for all scans is perpendicular to the core axes.

Specimens MIU-3/8 and MIU-3/10 are fine- to medium-grained biotite granites. CT scans were completed on both MIU-3/8 (Fig. 1A) and MIU-3/10 (Fig. 1B) at 15 μm resolution. For MIU-3/8, the pore sizes are smaller and less connected than in MIU-3/10 and are distributed in several “clouds” as well as in some planar features. Void space did not correlate with high-density phases (e.g. uranothorianite, zircon). MIU-3/10 displayed several large voids in the volume analysed but again pore space and high-density phases showed no positive correlation. (Pore volumes and additional CT scans are presented in the Supplementary Table 3 and Supplementary Fig. 1, respectively). Careful segmentation and imaging of calcite as a discrete phase reveals how this secondary precipitate has dominated the evolution of pore space within MIU-3/10. Supplementary Fig. 1F shows the distribution of calcite throughout the core volume, comprising 19.2% of the volume. A semi-transparent view of calcite shows that this phase is not monolithic (Supplementary Video 1, explained in Supplementary Note 3), but occurs as many small precipitates, filling in void space and decreasing the pore volume for MIU-3/10 relative to MIU-3/8. An additional scan at 7 μm resolution of MIU-3/10 (Fig. 1C) proximate to the main fracture was also completed, displaying pores with length scales of 50 microns or larger. At this scale there also does not appear to be a strong positional dependence on pore distribution.

CT scanning therefore gave two clear results. First of all, no obvious relationship between pores and high-density phases can be resolved, indicating that the imaged high-density phases are primary, and that the secondary phase assemblage does not typically include high density phases such as uranothorianite. Hence uptake of actinides is most likely by adsorption or incorporation in secondary minerals by co-precipitation of grains smaller than 7 μm Secondly, the CT scans show that MIU-3/10 has extensive primary phase alteration and secondary precipitation extending at least 10 mm perpendicular to the fracture. Secondary calcite occupies a significant fraction of the volume of MIU-3/10 near the fracture. Secondary precipitates follow parasitic fractures, clog pores, and may represent important sinks for trace elements that have been transported through conduits.

For comparison to the Toki Granite specimens we completed similar analyses on a sample from the Carnmenellis Granite. The Carnmenellis Granite specimen is broadly comparable (grain size, mineralogy, fracture geometry) to the Toki Granite specimens, however it was chosen as a comparator because it displays minimal alteration proximate to its main fracture, and hence represents a more pristine crystalline rock than either Toki specimen. We collected data covering all of the tests we completed on the Toki samples, but to extrapolate our findings concerning the length scale of RMD, the most useful data for comparison are the X-ray CT data. Figure 1D presents the pore (or void) space distributions for the Carnmenellis Granite. This core does not display any pervasive network of pores. Pore volume fractions for all specimens are given in Table S3: all of these are below 1% (note that the MIU-3/10 high resolution scan has a lower pore size threshold and hence exhibits higher void space fraction when compared to the lower resolution scan). The less altered Toki sample MIU-3/8 has the highest pore volume out of all specimens analysed by X-ray CT, however these pores are unconnected at this scale of observation. In Fig. 1D the Carnmenellis specimen has a “cloud” of void spaces similar to MIU-3/8, although again these are unconnected. Mineral alteration of the Carnmenellis sample is minimal, at most reaching microns away from the main fracture. The CT void space images of these two different lithologies clearly show that RMD length scales may be severely limited in crystalline rock, on the order of millimetres, because porosity is limited. The limits on porosity may be due either to initial limited void space or infill of porosity by secondary minerals.

### Fracture analysis and mineral alteration textures

**Figure 2:**
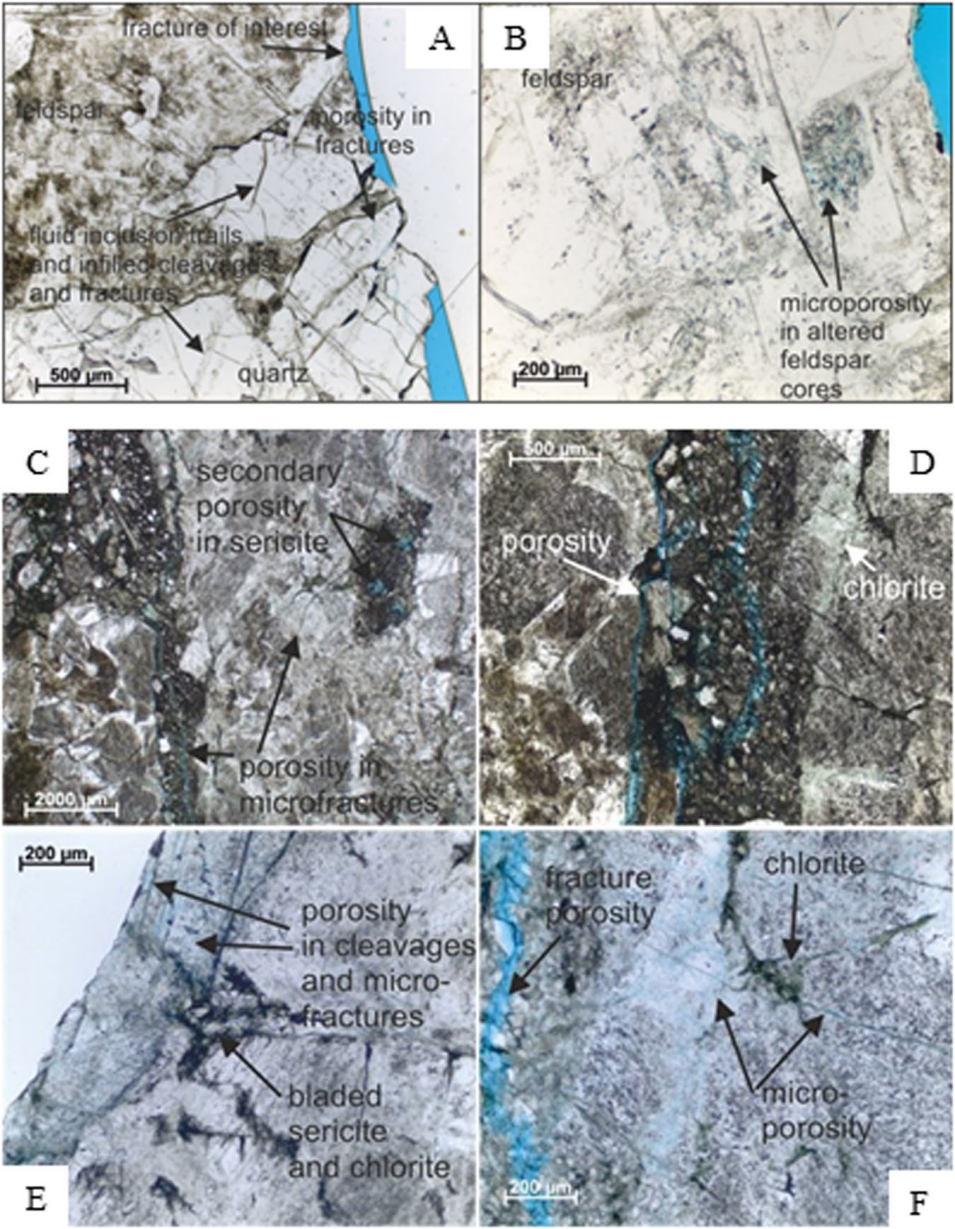
Optical photomicrographs of MIU3-8 (A,B) and MIU-3/10 (C-F) (proximal to main fractures) thin sections in plane polarized light (PPL). (**A**) MIU3-8 low magnification view including the fracture of interest, showing the altered nature of the alkali feldspars (turbid) and quartz crystals (clear) which contain numerous fluid inclusion trains, and cleavages and fractures infilled with a dark brown material (clay). (**B**) MIU3-8 plagioclase crystals showing altered cores. The alteration has led to the development of secondary microporosity. (**C**) Low magnification image of MIU-3/10 showing open fractures within the cataclasite in the centre of the thin section, and secondary porosity in the corroded core of a plagioclase. (**D**) Higher magnification of part of the MIU-3/10 cataclasite showing numerous associated open fractures. (**E**) Image showing the main fracture (blue dashed line) and vicinity in MIU-3/10. Porosity exists along the edge of this fracture in cleavages and microfractures. Some microporosity is also present in the chlorite and bladed sericite infill between grains. (**F**) High magnification view of microporosity in a sericitised and chloritised grain within MIU-3/10.

After constraining void space and mineral distributions throughout the core volumes near to main fractures, microfracture densities and changes to rock textures in the wallrock adjacent to the main fractures were examined using optical microscopy and BSEM-EDXA. MIU-3/8 is bounded by two planar main fractures inclined at 40–45° relative to the core axis. The matrix has a weakly sheared fabric, with numerous microfractures parallel to the main fracture surfaces. MIU-3/10 has an upper surface which is bounded by a steep fault inclined at about 10–20° to the core axis. A pale grey-green, calcite cemented, clay-rich, fault gouge/fault breccia is bound to the fracture wall. The wallrock matrix is strongly sheared with numerous microfractures parallel to the fault plane. The main fracture surfaces of both samples are coated with minor amounts of late euhedral calcite, a criterion for demonstrating open fracture porosity^30^. Optical photomicrographs of MIU-3/8 and MIU-3/10 are shown in Fig. 2. Both samples are bounded by fractures which coincide with zones of active groundwater flow in the borehole. In both cases there is alteration associated with microfracturing of the rock matrix and the development of networks of micro-porous grain boundaries and cross-grain micro-fractures. MIU-3/8 has less extensive fracturing, with parasitic microfractures extending into the wallrock for 5–10 mm from the main fracture. The microfracturing in MIU-3/10 is more complex, with evidence of early high temperature and late stage low temperature mineralisation. Proximal to the main fracture, MIU-3/10 displays intense parasitic fault shearing with crushed and comminuted wallrock extending for up to 10 mm from the main fracture. This cataclased wallrock has been heavily altered to a fine matrix of calcite, sericite, chlorite and probably smectitic clay. Micro-fractures extend into the MIU-3/10 wallrock from the main fracture for ≥20 mm.

**Figure 3:**
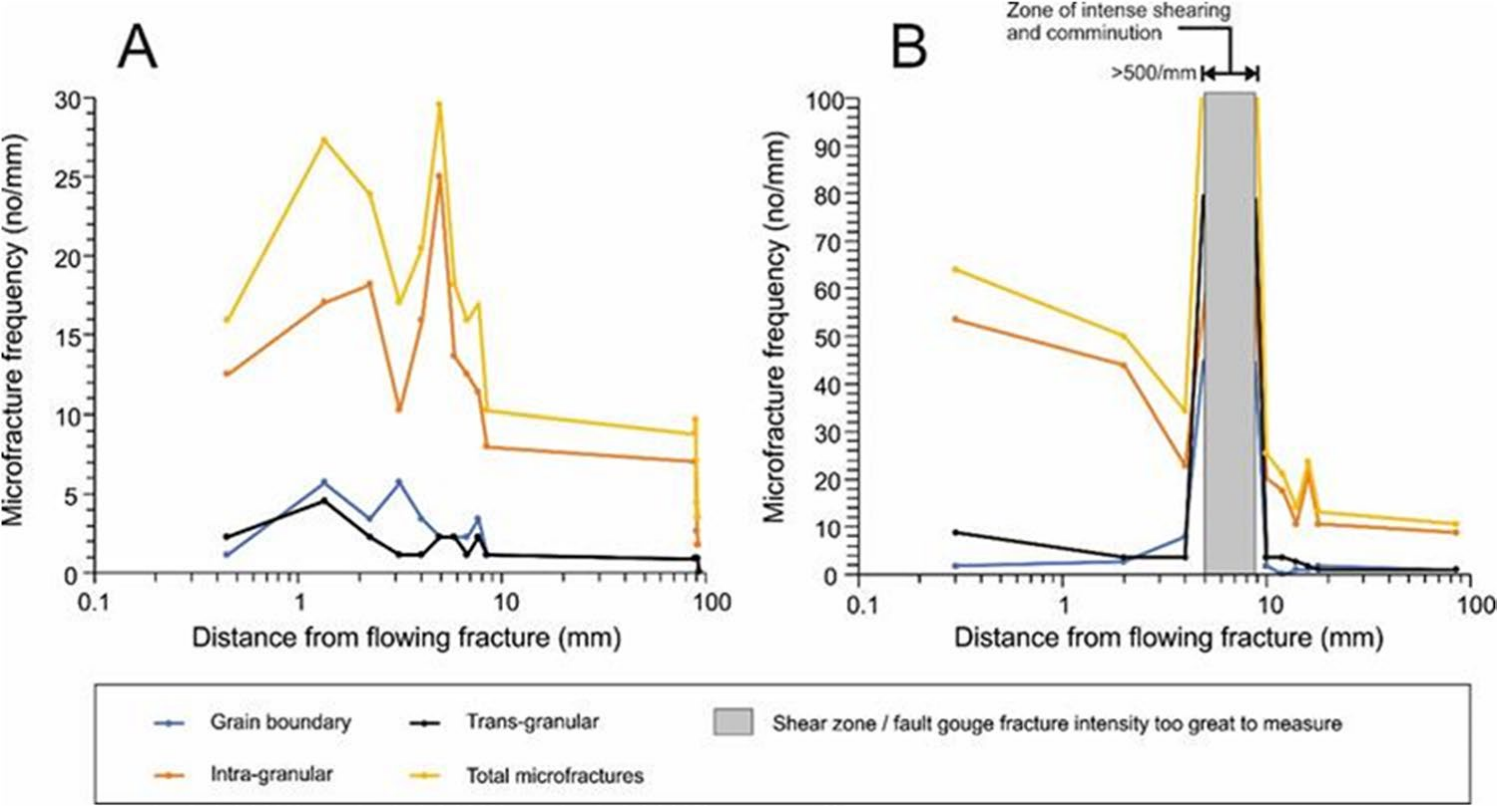
Distributions of microfracture types and total microfracture frequencies. (**A**) Transect perpendicular to the main fracture in MIU-3/8, estimated from backscattered scanning electron microscopy (BSEM). (**B**) Transect perpendicular to the main fracture in MIU-3/10, estimated from BSEM.

The distribution of microfractures in the wallrock adjacent to the primary fracture in these two samples is shown in Fig. 3 (measured as the frequency of fracture intersection along a scan-line taken perpendicular to the main fracture). These results will be biased towards features with orientations perpendicular to the scan-line and underestimate features parallel to, or intersecting the scan-line at acute angle. However, qualitative petrographic assessment showed that most transgranular features were sub-parallel to the main shear fracture. Sample MIU-3/8 displays a high density of fractures immediately adjacent to the primary fracture: up to 30 microfractures per millimetre decreasing sharply beyond 10 mm to a background level of about 10 fractures per millimetre. Most porous microfractures however are intra-granular (Fig. 3A). Trans-granular and grain boundary microfractures are subordinate but link with the intragranular microfractures to form an interconnected microfracture network. Microfracture apertures vary between <0.1 to 5 μm but are typically <2 μm. Additionally, there is significant secondary intra-granular microporosity hosted within plagioclase crystals caused by partial dissolution of the more calcic cores. Fluid access to the more calcic cores of the plagioclase crystals has been provided via the cleavage microfracture network and subsequent dissolution has proceeded along the feldspar cleavage planes. The microfractures and the dissolution porosity may be partially cemented by late calcite (see also below, where similar features are described for MIU-3/10). Calcite in the matrix is more abundant close to the main fracture, indicating that the mineralising fluid has permeated into the rock matrix from the main fracture. Calcium may be contributed locally to the fluid from breakdown and dissolution of plagioclase.

MIU-3/10 is highly fractured and sheared, with extensive wallrock alteration adjacent to the primary fracture. The wallrock is cut by a narrow zone, up to 4 mm wide, within which intense fracturing and shearing is associated with comminution and granulation of host rock material to produce a fine cataclasite. This shear zone begins 5 mm into the wallrock away from the primary fracture and is roughly 5 mm in width (Fig. 3B). The rock matrix between the primary fracture and the shear zone is very highly fractured with between 35 to 65 porous microfractures per millimetre (Fig. 3B). The microfractures are dominatly intra-granular but are intersected by a significant number of trans-granular fractures to form an interconnected microfracture network in the wallrock. Plagioclase crystals are particularly highly microfractured, especially along mineral cleavage planes, and most of the porous intra-granular microfractures are hosted within this phase. Grain boundary microfracturing is only a relatively minor feature of the wallrock.

Microfracture abundance increases dramatically within the narrow cataclasite zone. Because of the fine grain size, it was impractical to either enumerate the fractures here, or to differentiate the microfracture type. The abundance shown in Fig. 3B is a minimum estimate (i.e. microfracture population is probably>100 per millimetre). The surface of each comminuted fragment is effectively a “fracture” surface. Porosity within this sheared cataclasite zone is best described as “inter-particulate”. However, “through-going” dilatant porous fractures are also developed along this feature and sub-parallel to the main fracture. Further into the wallrock, beyond this shear zone, the fracture frequency decreases sharply to a background of 10 to 15 microfractures per millimetre at about 20 mm from the primary fracture (Fig. 3B).

As in MIU-3/8, the wallrock of MIU-3/10 also contains significant intra-crystalline microporosity resulting from the dissolution of calcic plagioclase crystal cores. Again, dissolution porosity is connected to the wider matrix porosity via a network of intra-crystalline microfractures. Some of the microporosity within the plagioclase contains fine grained secondary clay-rich (possibly smectitic) alteration products. The rock matrix also hosts abundant healed, or filled, microfractures. These healed microfractures are clearly early hydrothermal features, and are cross-cut by the porous microfractures and later calcite veinlets.

In MIU-3/10 microfractures partially filled by calcite and calcite veinlets are particularly common in the highly-fractured wallrock immediately adjacent to the main fracture. However, the extent of calcite mineralisation decreases further away from the main fracture. Calcite-mineralised, transgranular and intra-granular microfractures are particularly common within plagioclase crystals, which generally tend to be much more fractured. In some cases, this late-stage calcite may also partially fill dissolution porosity in the corroded calcic cores of the plagioclase. Similar features were also seen in sample MIU-3/8.

The presence of mineral cements (calcite) and phase alteration (plagioclase) indicates that processes of both dissolution and precipitation have modified these rocks in regions proximate to the fractures. Regions of extensive microfracturing extend no further than 20 mm from the main fracture, and alteration textures tend to be concentrated in a region also similarly proximate to the main fracture. In order to quantify the extent of the observed alteration reactions and also to discern the details of element transport, we completed detailed chemical analyses of wall rocks and fracture fills.

### Chemistry of wall rocks and fracture infill

ICP-OES data show that the extremely high concentration of Ca in the fracture material from MIU-3/10 far exceeds the expected Ca heterogeneity in the bulk rock, and is consistent with XRD and other analyses of this specimen (see Supplementary Fig. 2, Supplementary Table 4 and following) which clearly show calcite precipitation within the fractures. (All ICP-OES and ICP-MS data are presented in Supplementary Table 5.) Indeed, the Ca concentration in the fracture material approaches the Ca content of pure calcite (31.4% in the fracture compared to 40.0% in pure calcite). In contrast, Sr appears to be depleted in fracture infill material by ~50% in both specimens (determined by both ICP-OES and ICP-MS) probably due to dissolution of the plagioclase feldspar (the least stable silicate phase in this system^31^) and the subsequent loss of Sr due to the destruction of this host phase. Borehole fluid analysis confirms that Sr is indeed present as a solute (Table S2). Yttrium however is strongly enriched in the fracture infill of MIU-3/10, suggesting that Y is incorporated into the secondary calcite preferentially after release from the primary phases (feasible given the observed correlation between Y and carbonates in sedimentary systems^32,33^).

There is also an interesting REE pattern in both Toki Granite samples. The fracture infill in the less altered sample (MIU-3/8) is depleted in all REE (see Supplementary Fig. 3). In the more altered specimen (MIU-3/10), the fracture infill is likewise depleted in the LREE (La, Ce, Pr, and Nd), but enriched in the HREE (Gd, Tb, Dy, Ho, Er, Tm, Yb, and Lu) as shown in Fig. S3B. Similar behaviour has been observed experimentally during calcite precipitation when REE element concentrations were extremely high in the reactant fluid^34^, and this implies that REE concentrations were high in the reactant fluids in contact with MIU-3/10.

ICP-MS data indicate that some of the primary phase Th and U may have been mobilized by fluid flow through these fractures, with Th and U depleted in the fracture infills for both MIU-3/8 and MIU-3/10 relative to the bulk rock (Table S5). This implies that they were mobilized as the primary phases were dissolved, but unlike Y they were not preferentially incorporated into secondary precipitates during formation within the fracture region. From these data we conclude that there has been important loss of LREEs, U, Th, and Sr from the primary minerals via mineral dissolution reactions. Precipitation reactions have in contrast produced phases enriched in HREEs and Y. Because U is especially mobilized by oxidising fluids, the breakdown of primary actinide-bearing phases and relative loss of U further implies that the reactive fluids in this system were sufficiently oxidizing that U(VI) was the dominant U oxidation state.

### Trace element maps constrain length scales of alteration

**Figure 4:**
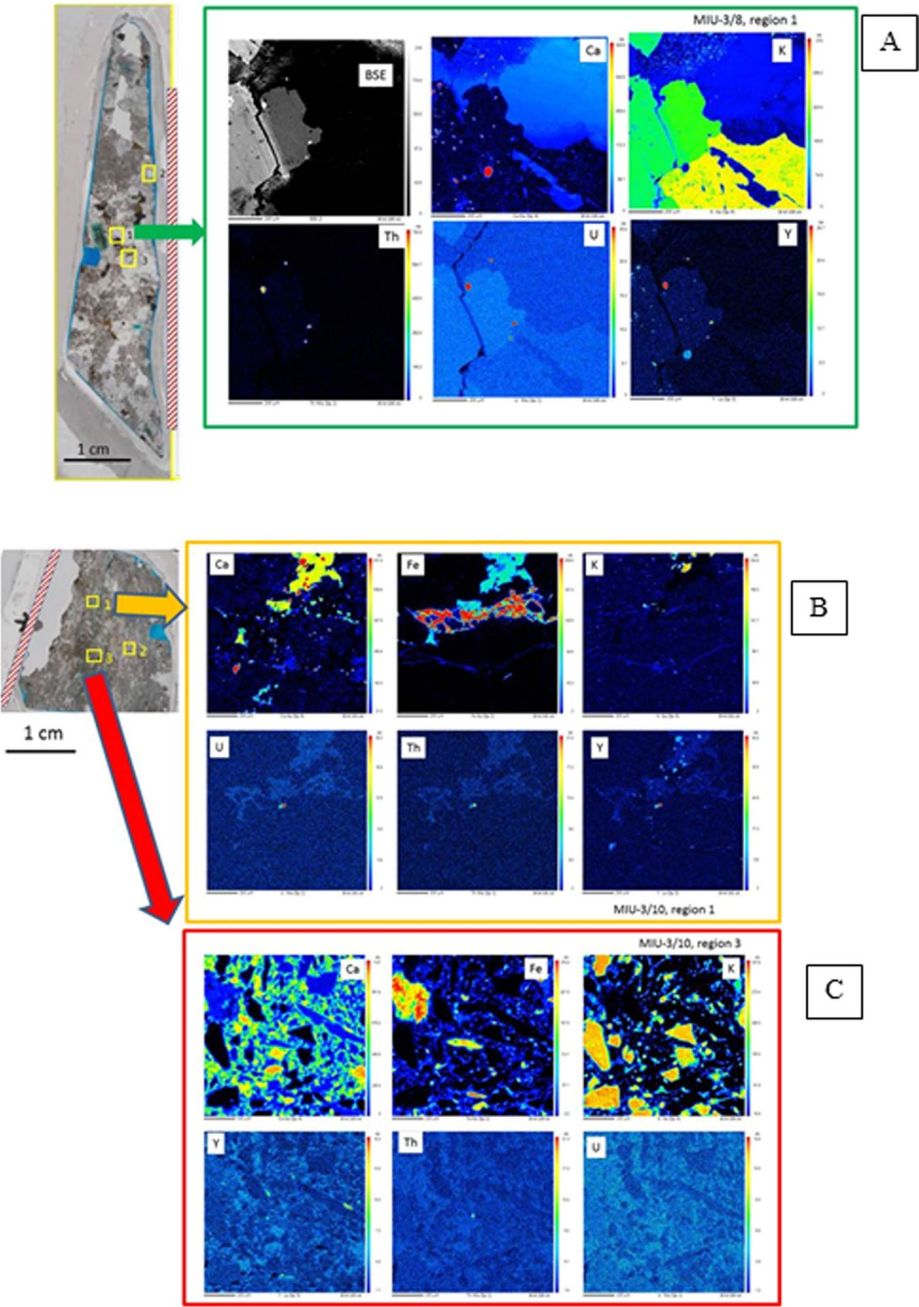
Electron Microprobe maps, with locations shown on the insets at left of each panel. False colour intensities correspond to the concentrations of the elements labelled in the panel, with red the highest relative concentration grading to blue at lowest concentrations. Each panel is 1 mm ×1 mm. Main fracture orientations indicated by red cross-hatched regions. (**A**) Map of MIU-3/8 approximately 6 mm from the main fracture. This is the most pristine area mapped by microbeam techniques. The small U and Th-rich grains are either apatite or actinide oxides. (**B**) MIU-3/10. Upper panel shows select major element (Ca, Fe, K) and trace element (U, Th, Y) distributions in a region 6 mm from main fracture. Calcite infill with secondary Fe-minerals is present. Y maps with Ca within the mineral calcite, and some small U/Th/Y hotspots are visible. (**C**) Parameters same as previous panel. Region is 8 mm from main fracture but directly on a parasitic fracture. Calcite infill dominates, with fragments of primary phases present. Y (and possibly U & Th) map with Ca in calcite.

Our ICP data provide interesting insights into possible mass transfer at Toki, however those data are averaged over all solid phases and have only coarse spatial resolution (wall rock vs. infill). Use of trace element chemistry to track mass transfer in this system requires knowledge of initial mineral compositions. Here we use several trace elements (i.e. Y, U, Th) to document the mobility of trace elements and constrain the length scales of mass transfer in the Toki specimens. Trace element maps obtained through electron microprobe analyses (EMPA) of the most pristine area in MIU-3/8 (Fig. 4A) show that Y is concentrated in large primary apatite grains, while Th and U correlate in small actinide oxides or zircons that occur in the boundaries of K-feldspar crystals. Major element EMPA maps of regions closer to the fracture clearly show plagioclase alteration and reveal high Th, U, and Y grains within those regions that exhibit nascent alteration. Grain boundaries and fracture edges near the main fracture in some cases display Fe-enriched via elemental mapping. This Fe-enrichment is most likely due to fine-grained precipitates of Fe-oxyhydroxides. In addition, small bright spots of Ca are resolved along the borders of large biotite crystals. These are most likely tiny calcite precipitates. Calcite is the dominant secondary phase in the more well-developed fracture in MIU-3/10, and therefore these small calcite precipitates in MIU-3/8 probably mark the first stages of similar rock alteration. Thus, there is clear chemical evidence for incipient surface alteration, plagioclase dissolution, and secondary precipitation reactions within 6 mm of the main fracture in MIU-3/8.

EMPA maps of Ca and Fe near the fracture in MIU-3/10 reveal significant alteration (Fig. 4B). High concentrations of Ca and Fe dominate the upper part of the images, with smaller grains of Ca and Fe phases distributed along microfractures through the remainder of the region. U, Th, and Y are associated with some of the small secondary grains. The element distributions and mineral textures here are completely different from MIU-3/8. In contrast, maps from the most distal region of MIU-3/10 much more closely resemble the image from MIU-3/8, with minimal alteration and no evidence of secondary phases (Supplementary Fig. 4).

Another area close to the main fracture in MIU-3/10 but straddling a parasitic infilled fracture is mapped in Fig. 4C. Here, primary phases are extensively broken down, with residual small grains of K-feldspar embedded within a background matrix of calcite and Fe-rich phases. Yttrium is concentrated into the calcite and correlates with Ca in the secondary infill. There is one hotspot of Th located within the altered region. Elemental linescans and maps show that alteration is strongly controlled not just by proximity to the main fracture, but by proximity to the small parasitic branching fractures.

Alteration of MIU-3/10 is significantly more advanced and pervasive than MIU-3/8, but the reaction pathways are parallel. Specifically, feldspar dissolution is occurring along with calcite and iron oxyhydroxide precipitation. U, Th, and Y provide useful indicators of mass transfer caused by fluid flux because there is an inventory of primary U, Th, and Y bearing phases from which these elements may be sourced but they also are detectable within highly altered regions; e.g. hotspots of discrete grains with high Th and U concentrations are resolved within secondary infill. In addition, Th, U, and Y also occur within secondary minerals at lower concentrations, apparently as co-precipitates within calcite. These concentrations are however at the detection limits of EMPA. Therefore, other measurements including microfocus synchrotron X-ray Fluorescence (XRF) mapping, X-ray absorption spectroscopy (XAS), autoradiography, and γ-spectrometry were completed to further constrain chemical inventories, bonding environments, and alteration length scales.

**Figure 5:**
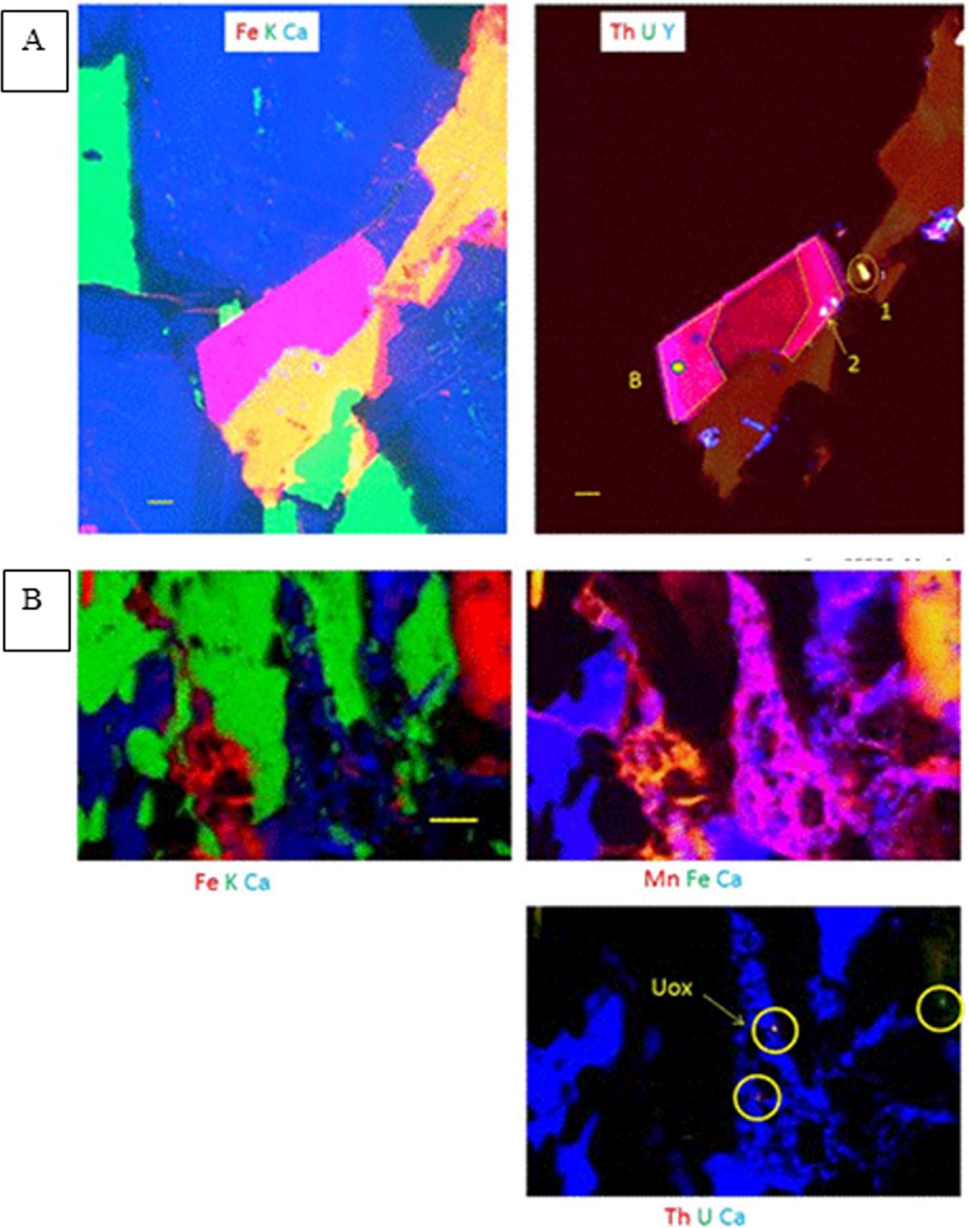
Synchrotron microfocus XRF maps. (**A**) MIU-3/8 Left: Fe (red), K (green), and Ca (blue). Minerals are as follows: green = K-feldspar, blue = plagioclase (mottled areas indicate plagioclase breakdown to phyllosilicate, especially in the more anorthitic cores), yellow = biotite, and pink = bastnaesite, a rare earth fluoride-carbonate, CeCO_3_(F). Right: Th (red), U (green), and Y (blue). This crystal of bastnaesite is rich in Th. Discrete grains of a U-rich phase appear as small yellow areas, while blue acicular regions are rich in Y and may be a REE/Y rich phosphate phase. Yellow dot labelled ‘B’ is approximate location of Th-XANES and bastnaesite point analysis. Points labelled 1 and 2 correspond to AcO_2_ point analyses 1 and 2 in Table 2. Th XANES was also taken at the point labelled 1. (**B**) MIU-3/10 synchrotron microfocus XRF map. This image includes mineral infill associated with secondary fractures. False colour elemental map, with colour key provided as element symbols coloured to correspond to map colours. Top left major element map shows K-feldspar in green, iron oxyhydroxide in red, and calcite in blue. In the top right panel, the magenta region is calcite with enriched Mn concentrations. Bright orange shows the incorporation of Mn into Fe-oxyhydroxides. The lower right panel clearly shows the calcite precipitates and small regions within the infill that have high concentrations of both Th and U (yellow circles). The hotspot labelled Uox indicates the location of the oxidized U L-III XANES spectrum presented below. 100-micron scale bar.

Microfocus synchrotron techniques were used to map chemical changes relative to mineralogical alteration, to determine actinide and lanthanide concentrations, and to investigate possible changes in co-ordination of U and Th as they migrated from primary to secondary minerals. Three regions of each specimen were mapped. Figure 5A, left panel illustrates the least altered part of MIU-3/8. Grain boundaries are sharp and mineralogy is dominated by primary phases. Biotite cuts across the field of view from the upper right (yellow), K-feldspar is (green) at left and bottom, and plagioclase dominates the assemblage (blue). Mottling indicates alteration of the feldspars, and again emphasises that cores of plagioclase grains are preferentially altered. Thus, some alteration is present throughout this specimen, even several mm from the fracture.

The pink crystal at the centre of Fig. 5A, is primary bastnaesite, a rare-earth fluoride-carbonate. The left panel shows that this phase contains both Fe and Ca, while the right panel shows that it is enriched in Th and Y. Maps of U and Th (right panel) also show that tiny actinide oxide crystals are associated with the bastnaesite. Both bastnaesite and actinide oxide represent primary host phases for actinides and lanthanides within the granite, and therefore they are the starting points for understanding mass transfer of these elements.

Point analyses were taken on several of these hotspots on MIU-3/8. Calculated concentrations for select elements are shown in Supplementary Table 6. These results show that there are at least three distinct lanthanide and actinide host minerals within the Toki Granite: bastnaesite, uranothorianite ([Th,U]O_2_), and zircon. Furthermore, the uranothorianite phase is quite variable in terms of the amount of U in the structure. These phases dominate the actinide and lanthanide inventories within the primary minerals of the MIU-3 specimens. Due to synchrotron time constraints we were not able to complete XRF point analyses on MIU-3/10.

Two more regions were mapped in order to accurately represent the distribution of major and trace elements in MIU-3/8. Hotspots of Th and U were particularly sought out. One scan (Supplementary Fig. 5) was completed near a large biotite crystal, where grains rich in both U and Th were imaged. Here, one large grain also contained relatively high Y concentrations, and a point analysis (see Supplementary Table 6) further highlighted the variable chemistry of the actinide oxides. Heterogeneity of the actinide oxides was additionally confirmed by mapping of a third area on MIU-3/8. Alteration of K-feldspar is clearly resolved in Supplementary Fig. 6 (left-hand panel, where K-feldspar in green is significantly mottled) while Th/U grains are again located proximate to a large biotite crystal (orange in the left-hand panel). Zircon was here identified as an additional primary host phase for actinides. A zircon point analysis was obtained (labelled in Supplementary Fig. 6 and contained within Supplementary Table 6). The best signal to noise XANES spectrum for U within MIU-3/8 was obtained from the area labelled ‘Ured’ as shown on Supplementary Fig. 6.

Synchrotron analysis of specimen MIU-3/10 investigated how alteration had affected the region proximate to the large active fracture. Locations were therefore analysed that had clear evidence of infill precipitation and in some areas infill precipitates even dominate the analysed volume. The left panel of Fig. 5B shows primary K-feldspar (green), but the remaining areas are either secondary calcite (blue) or Fe-oxyhydroxide (red). Mn occurs within both calcite and Fe-oxyhydroxide (Fig. 5B, upper right panel) which suggests precipitation of carbonate during fluctuating redox conditions because Mn(IV) is typically co-precipitated with Fe in hydroxide phases but only reduced Mn(II) is compatible with calcite. Perhaps most importantly, the maps of Th and U (lower right panel) show clear hotspots of actinides within the fracture infill (indicated by yellow circles). Actinide-enriched hotspots were relatively common within the altered infill of MIU-3/10 (e.g.: circled areas on Fig. 5B) and the best quality U XANES spectrum from within the infill was obtained here from the grain labelled ‘Uox’. An additional elemental map (Supplementary Fig. 7) shows thin sinuous precipitates of calcite filling microcracks, diffuse films of Fe-oxyhydroxides, and a small crystal with extremely high Th content proximal to the calcite infill. A lower resolution ‘overview’ map (Supplementary Fig. 8) confirms that large fractured K-feldspar crystals (upper left map, green) have secondary calcite precipitates running the length of the fracture (blue). Within the calcite infill a number of Th-rich grains can be resolved, strongly correlated with the calcite minerals (lower right panel). Mn is present within some of the calcite, along with Y, and here Sr also is apparently somewhat enriched within the carbonate (white areas in the upper right panel).

X-ray absorption near edge spectroscopy (XANES) spectra of U and Th constrain coordination chemistry and thereby help understand the attachment processes governing actinide mass redistribution within these fractures. Supplementary Fig. 9 shows a comparison of U L_III_ XANES spectra. The blue curve is from a uranium-rich microcrystalline phase in specimen MIU-3/8 relatively distal from the main fracture and inferred to be primary (see Supplementary Fig. 6). The red curve is from a uranium-rich microcrystalline grain in MIU-3/10 in a region of intense micro-fractures (see Fig. 5B above). Using the Greaux^35^ methodology, the edge position of the U in MIU-3/8 is determined to be at 17166.7 eV, essentially equal to the edge position of fully reduced U(IV). However, the spectrum for the U within the fracture region of MIU-3/10 is clearly displaced to higher energy (horizontal arrow). In this case, the edge is determined to be at 17168.4 eV, an energy shift of +1.7 eV, approximately equal to 60% of the theoretical energy difference between fully reduced and fully oxidized uranium. Furthermore, a shoulder appears on the high energy side of the MIU-3/10 spectrum (vertical arrow) which is not present on the MIU-3/8 data. This high energy shoulder is a typical feature of a U(VI) XANES spectrum^36^. Together, the positive edge shift and appearance of the shoulder feature indicate that uranium in secondary phases from the MIU-3/10 fracture zone is significantly oxidized relative to uranium in primary phases. Because the edge position in MIU-3/10 is intermediate between U(IV) and U(VI), the uranium within the fracture is clearly on average of higher oxidation state than in the pristine host minerals but probably includes a mixture of different valencies. We note however that the oxidized U(VI) component determined via XANES within the carbonate vein-associated uranium hotspots on MIU-3/10 is not compatible with typical Ac-La valency within any of the primary actinide-bearing phases, and therefore the oxidized U near the fracture in MIU-3/10 must either be within an altered surface layer or is associated with a different mineral, most likely a secondary mineral precipitate such as calcite. XANES spectroscopy of Th is presented in Supplementary Fig. 10.

In summary, synchrotron XRF microfocus imaging has mapped the distributions of Th, U and Y in primary mineral phases within the less-altered specimen MIU-3/8 and XANES spectroscopy showed that uranium here is reduced U(IV). In contrast, U and Th are correlated with secondary carbonates and other phases associated with fracture infill within the more altered specimen MIU-3/10. XANES spectroscopy of uranium within the fracture infill showed significant oxidation: either the hotspots of U within the fracture infill are oxidized remnants of the primary actinide host phases, or, more likely, these are secondary phases which have incorporated small quantities of actinides that were mobilized by oxidative dissolution of primary phases upstream of this specimen. It has been shown experimentally that U(VI) will adsorb onto alkaline earth oxides and carbonates^36^.

These results strongly imply that some fraction of primary phase U(IV) was mobilized and lost from the parent rock by oxidative dissolution, with trace quantities of the mobile U(VI) incorporated into secondary carbonates as they precipitated as part of the fracture infill. These analyses also indicate that Y has been mobilized from primary phases but then preferentially concentrated into secondary calcite.

**Figure 6:**
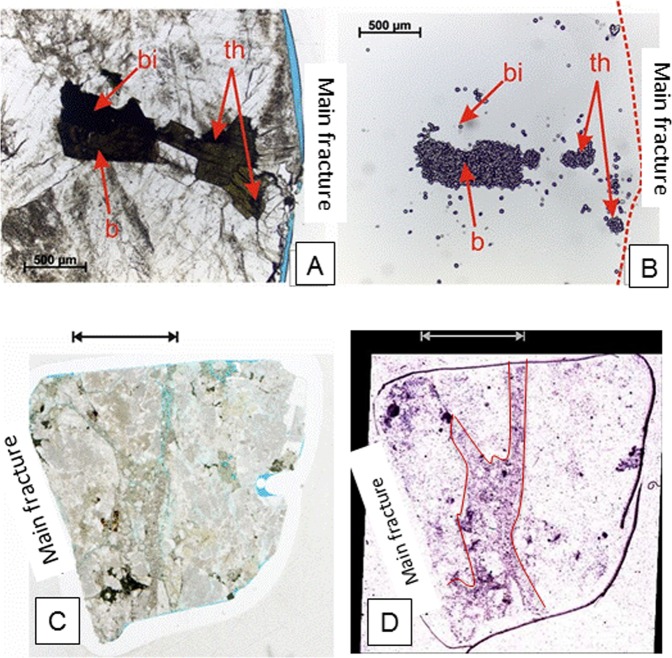
α-radiography of Toki granite specimen thin sections taken near the vicinity of the primary fracture. (**A**) Toki Granite sample MIU-3/8 - transmitted light photomicrograph of wallrock adjacent to the calcite-encrusted surface of the main fracture, showing a primary bastnaesite crystal (b) associated with an aggregate of biotite laths (bi). (**B**) α-autoradiograph showing a very high density of α-tracks corresponding to the bastnaesite crystal (b) and to discrete small crystals of uranothorianite (th) occurring as inclusions in biotite. Low concentrations of α-tracks can be seen to correspond to the grain boundaries of the biotite and along microcracks in the adjacent plagioclase feldspar. (**C**) Toki Granite sample MIU-3/10 A: Transmitted light scanned image of whole polished thin section. (**D**) Alpha-autoradiograph of the whole polished thin section. This autoradiograph shows intense but diffuse α-radioactivity closely associated with the highly fractured and cataclased wallrock within a clay-rich alteration zone (indicated by arrow) extending up to 10 mm into the host rock from the main fracture (region of enhanced α-activity approximately outlined in red). Scattered, discrete highly radioactive ‘hot-spots’ are distributed across the section and are associated mainly with bastnaesite, and accessory uranothorianite, zircon and apatite that occur as inclusions within biotite, allanite and bastnaesite. Field of view of thin section = 28 mm wide.

Further mapping via α-autoradiography revealed that the main fractures in these two samples have influenced the distribution of α-radioactivity in the wallrock (Fig. 6). In MIU-3/8 (Fig. 6A), the radioactivity is decreased in a narrow zone immediately adjacent to the fracture (Fig. 6B), extending for 2–4 mm into the wallrock. This indicates loss of uranium and/or thorium from the wallrock within this zone. Sample MIU-3/10 shows markedly different behaviour (Fig. 6C). The altered wallrock shows an enhancement of α-radioactivity relative to unaltered rock (Fig. 6D). The primary sites of α-radioactivity in the host rock are uranium and thorium-rich minerals, including igneous uranothorianite, bastnaesite, zircon, monazite and apatite. However, in the altered wallrock, these minerals are being broken down, with a concomitant reduction in their α-activity. This indicates the leaching of U and/or Th from these phases and is completely consistent with the mass transfer indicated by ICP-MS and synchrotron analyses. However, the altered wallrock in MIU-3/10 appears to be much more α-radioactive compared to both the relatively unaltered background rock in MIU-3/10 and the bulk and altered specimens sampled from MIU-3/8. We supplemented the radiography images with γ-spectrometry to determine the source of the elevated α-activity in the fracture infill in MIU-3/10.

Peaks associated with U, Th, Pb, and Ra were unequivocally detected via γ-spectrometry (see Supplementary Fig. 11). The data clearly show that the enhancement in γ–ray activity at 609 keV in the MIU-3/10 samples is due to Ra uptake into the calcite. The actinide activity is consistent with all of the other analytical work on these specimens, and the definitive identification of ^226^Ra provides support to the hypothesis that the enhanced radioactivity in the highly altered wallrock in sample MIU-3/10 observed in the auto-radiography data is due to Ra introduced via fluid flow. This is supported by JAEA’s previously published results from MIZ-1^25^. As an alkaline earth element, Ra is compatible with the crystal structure of calcite and could be taken up by calcite precipitation in high enough amounts to produce the auto-radiographs presented above. Radium concentration within the more altered fracture region of MIU-3/10 (5.74 ×10^–12^ g ^226^Ra/g granite) is nearly three times higher than that in the comparable area of MIU-3/8 (1.94 ×10^–12^ g ^226^Ra/g granite). This is another clear chemical indicator of mass transport associated with the fractures.

Chemical mapping and spectroscopy considered together show that there has indeed been mass transfer of radionuclides and other solutes associated with the main fracture. The presence of oxidized Fe, Mn, and U in secondary minerals indicates that the reactant fluids must have been oxidizing for at least portions of the reaction path history. Furthermore, since the unaltered host rock is low in carbonate, the most likely source of carbonate in the precipitated calcite is the fluid phase. Part of the chemical inventory mobilized through dissolution of the primary phases does become sequestered within the secondary infill precipitates. In the case of Y and Ra the infill precipitate becomes significantly enriched in these elements, whereas for U and Th the uptake from solution is low. Analysis of the fluid in the borehole is wholly consistent with the mineralogical and geochemical data: the fluid contains high enough bicarbonate levels to be supersaturated with calcite at ambient T and pH (Supplementary Table 2), and the presence of sulfur as a sulfate species confirms the presence of a relatively oxidized fluid. While all of the chemical data indicate mass transport along the main fractures, they also place unequivocal constraints on the length scale of reactivity perpendicular to the fracture. For both specimens the length scale of fluid access is small, as defined by the chemical processes of trace element gain/loss, primary phase breakdown, and secondary phase precipitation. For MIU-3/10 the length scale ≤ 20 mm, while for MIU-3/8 this value is ≤ 6 mm. These length scales are comparable to those determined by void space, microfracture density, and textural analysis.

## Discussion

This study provides constraints on the movement of fluids and dissolved chemical species in and around fractures in the Toki Granite. The heavily altered sample (MIU-3/10) shows evidence that the rock matrix has taken up ^226^Ra from water flowing through the main fractures. This uptake would most likely have occurred within the last 8,000 years since the half-life of ^226^Ra is 1,600 years and after 8,000 years over 95% of the ^226^Ra would have decayed. Trace element loss of U, Th, and Sr was also demonstrated in the Toki samples, through removal from primary minerals in the wallrocks of fractures as they altered and dissolved. However, not all of the mobilized primary trace element inventory has been removed from the system, because some of the U, Th, Sr, and especially Y that was mobilized from primary minerals has been fixed within secondary mineral precipitates (primarily calcite but also clay minerals and iron oxyhydroxides) within the fractures.

2D visualisation of the porosity, based on the imaging of the distribution of blue-dyed epoxy resin impregnation by optical and backscattered SEM, together with 3D X-ray CT imaging of void space showed the pores to have spatially limited connectivity. There is no evidence that water has penetrated from the main conductive fractures into the rock matrices for more than a few centimetres, even in the most altered sample. Microfracture networks have provided pathways for the migration of trace elements but are spatially related to major fractures that are believed to support active groundwater flow. With increasing distances from these latter fractures, the frequencies of the microfractures drastically decrease.

Transport, retardation and alteration processes are closely coupled. The spatial distributions of the studied fractures and their related microfractures may be related to larger fractures that dominate advection of groundwater and are spatially related to mineralogical and chemical alteration, probably due to water being able to access the rock matrix via the micro-fractures. This process then allows a proportion of natural radionuclides and other trace elements to be mobilized from primary minerals in the rock matrix as they break down.

**Figure 7:**
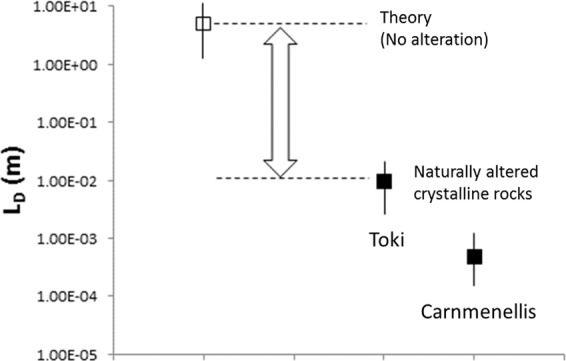
Conceptual reassessment of the effective length scale of rock matrix diffusion. In low temperature systems within crystalline rock the results presented above provide evidence showing that mineral alteration and trace element transport constrain the length scale over which RMD will operate (L_D_), and in natural rocks affected by alteration it is shown to be 2 to 5 orders of magnitude smaller than the commonly proposed values.

A key result is that the effective length scale of rock matrix diffusion, as recorded by chemical changes to the rock, is small in the specimens analysed. Figure 7 compares the length scale of diffusion as directly measured in this study. In the case of the most reacted specimen (Toki Granite MIU-3/10), a large volume of secondary mineral precipitates, mostly calcite, has filled in void space to produce a set of pores that are volumetrically smaller than in the less altered specimens and of decreased connectivity. This specimen may have had a much larger transient porosity prior to growth of the infill.

Consideration of the volume changes which occur during dissolution of the primary phases in granite and subsequent precipitation of secondary phases from water, especially a fluid that has a significant inventory of dissolved carbonate, explains why rock matrix diffusion may be less effective in some more reactive systems than in systems with little or no mineral reactivity. Direct analysis shows that the fluids flowing within the Toki Granite hydrologic system are supersaturated both with calcite and magnesium-aluminium silicates (Supplementary Table 2). This is consistent with the secondary phase assemblage we observe here. Based on all of our observations, we may write an overall balanced alteration reaction:

$$3\,{\rm{A}}{\rm{n}}{\rm{o}}{\rm{r}}{\rm{t}}{\rm{h}}{\rm{i}}{\rm{t}}{\rm{e}}+2\,{\rm{P}}{\rm{h}}{\rm{l}}{\rm{o}}{\rm{g}}{\rm{o}}{\rm{p}}{\rm{i}}{\rm{t}}{\rm{e}}+3\,{{\rm{H}}{\rm{C}}{\rm{O}}}_{3}^{-}+5{{\rm{H}}}^{+}\to 3\,{\rm{C}}{\rm{a}}{\rm{l}}{\rm{c}}{\rm{i}}{\rm{t}}{\rm{e}}+{\rm{C}}{\rm{l}}{\rm{i}}{\rm{n}}{\rm{o}}{\rm{c}}{\rm{h}}{\rm{l}}{\rm{o}}{\rm{r}}{\rm{e}}+3\,{\rm{Q}}{\rm{u}}{\rm{a}}{\rm{r}}{\rm{t}}{\rm{z}}+2\,{\rm{S}}{\rm{e}}{\rm{r}}{\rm{i}}{\rm{c}}{\rm{i}}{\rm{t}}{\rm{e}}+{{\rm{M}}{\rm{g}}}^{2+}$$

This reaction captures the following facts: (1) biotite (here represented as phlogopite) and plagioclase (anorthite) are the most reactive primary silicates, (2) dissolution should be an acid-consuming reaction, also consuming water, and (3) carbonate must be introduced from the fluid phase. It also reflects the secondary assemblage identified in this study including calcite, clinochlore, quartz, and sericite. This reaction shows a large positive ΔV, equal to a > 10% solid phase volume increase over the initial assemblage (see Supplementary Note 2 for details on this calculation). There are many alternative ways to write this reaction, however from the constraints placed on the calculation by the observations, the positive ΔV is clearly a result of the fact that this is not just a conversion of primary silicates to more hydrated phases, but also includes growth of secondary calcite which accounts for most of the solid volume increase. Therefore, in granite and other crystalline rock fracture systems where fluids exceed secondary phase supersaturation, it is most likely that alteration will decrease pore network volumes and connectivity as clearly observed for these Toki Granite samples. Shutting down of fluid transport through mineral precipitation is a well-known problem in the hydrocarbon industry, where flow in even a large aperture pipe can be completely cut off by mineral scale formation in 24 hours^37^. This process of secondary precipitation may act to limit rock matrix diffusion to short length scales, as seen here. Furthermore, most of the observed alteration takes place in what we shall refer to as a “damaged zone” around the main fracture. The development of this damaged zone provides a major constraint on the ability of fluids to penetrate into the bulk rock mass. Away from the damaged zone, diffusion through the less fractured matrix may be strongly inhibited if the matrix pores are not already water-saturated. From this we postulate that if a host rock is undergoing uplift, and is progressively invaded by meteoric water, the effectiveness of RMD will decrease as a function of time. This raises the question as to whether radionuclides or other solutes might enter the rock matrix by RMD early in uplift and then become “sealed in” to the rock matrix later in uplift owing to the processes highlighted here.

Our most important conclusion is that there is a need to change the conceptual understanding of the process of rock matrix diffusion over timescales that are sufficiently long for transport properties to evolve. In the present study, no evidence for extensive (more than a few cm) RMD from flowing fractures has been found. Instead, it is apparent that transport properties may change as mineralogical and structural alteration proceed. Therefore, a single diffusion coefficient cannot be used to explain diffusion throughout the rock matrix because the damaged zone has different hydrogeological properties and precipitation reactions will dynamically act to decrease fluid access to pores. Furthermore, in granite the preservation of primary igneous mineral phases beyond the observed length scales of alteration strongly implies that the distal part of the rock matrix is not accessible to water, since many of these mineral phases are thermodynamically unstable in the presence of water (e.g. biotite).

Previously our understanding of rock matrix diffusion could be summarized by six fundamental concepts.

1. Advection of groundwater occurs through major, interconnected fractures.
2. Transport of water and solutes may occur by advection extending for short distances into the wallrocks of major fractures, especially into a “damaged zone” where the wallrocks develop connected porosity.
3. Dissolution of primary phases may act to increase void space.
4. The frequencies, sizes, connectivities and tortuosities of pathways comprised of the two kinds of pores (micro-fracture porosity and secondary dissolution porosity) are related, since connected micro-fractures provide pathways for water and solutes that are necessary for mineral alteration / dissolution, and for water that removes solutes released by these reactions.
5. The proportion of transport that occurs by advection diminishes with increasing distance from the main fracture.
6. The proportion of transport that occurs by diffusion increases with increasing distance from the main fracture^38^. However, as shown in Fig. 7, this conceptual model for rock matrix diffusion in crystalline rock needs to be updated to reflect the fact that the length scale of this process may be severely limited.
  The present study adds five new additional concepts:
7. Damage zone porosity is a combination of micro-fracture porosity (involving fractures with a range of spatial scales) and secondary porosity due to mineral alteration / dissolution, however there will likely be poor initial connectivity of micro-pores.
8. If the fluids are supersaturated with carbonates and clay minerals, even if dissolution of the primary minerals is occurring, pore space may DECREASE significantly as a function of reaction progress because the Δ even if dissolution of the primary minerals is occurrielative rates of silicate dissolution (slow) vs. carbonate precipitation (fast) favour secondary phase formation.
9. The frequencies, lengths, apertures and connectivities of the microfractures diminish rapidly (perhaps exponentially) with increasing distance from the main fracture.
10. The intensity of mineral alteration/dissolution also decreases rapidly with increasing distance from the main fracture.
  Finally, because we have demonstrated a close link between mineralogical reactions and the incorporation of actinides, lanthanides and other trace elements into fracture wall rocks we can make one additional point. Together with the evidence for enhanced transport in the fracture damaged zone, our results imply that solute species introduced in infiltrating fluid or released from primary phases could be incorporated into the altered wall rocks at a rate determined by the rates of mineral precipitation reactions, not by the rate at which they are introduced. Therefore, we add:
11. The fixing of such solutes into wall rocks, commonly thought of as RMD, is actually limited by mineral reaction rates and not by diffusion, meaning that it may be a relatively inefficient process in terms of its effectiveness at fixing solutes even before the limited spatial extent of fluid penetration is taken into account.

Within the sections of wallrock through which some advection is possible, there is still the possibility for some solute retardation by diffusion into parts of the rock between connected micro-fractures, which also have limited connected secondary porosity. Beyond a certain distance from the main fracture, transport of water and solutes will occur dominantly by diffusion. This distance will depend upon the nature of fracturing in the wallrock of the main fracture (frequencies, apertures, connectivities etc.), which in turn will depend upon the lithology and its deformation/alteration history. In other words, the distance over which RMD operates will be locality-specific, potentially even at the borehole-scale.

Transport of water and solutes, even by diffusion, will most likely become insignificant beyond a certain distance from the main fracture through which advection occurs. Possibly, this distance may be marked by an alteration front. The micro-porosity beyond a certain distance from the main fracture (further, at least, than any alteration front) may not be water-saturated. Mineral alteration/precipitation/dissolution (which is spatially related to micro-fracturing) will influence the ability of the rock to retard solutes (e.g. radionuclides), either by sorption, or by precipitation/co-precipitation. Some reactions between water and rock will tend to release solutes to the aqueous phase, while other reactions will tend to sequester solutes. The elements U, Th, Sr, and Y are effective indicators of alteration and mass transport. Uranium speciation and Fe-oxyhydroxide phases serve as indicators of fluid redox state, and the presence of carbonate minerals (or similar precipitates) may provide a useful index for deciding whether RMD is likely to be effective in a crystalline system: e.g. if carbonate precipitates are present then RMD will almost certainly be restricted to short length scales.

We conclude that that the length scale on which RMD will operate on in crystalline rock is site-specific. It seems unreasonable to assume that the process of RMD is necessarily occurring in a crystalline rock without site-specific evidence. Even over timescales of many thousands of years, RMD may occur over length scales of millimetres to centimetres.

## Methods

### X-ray radiography

The whole-core samples were initially examined by X-ray radiography which provided information about the characteristics of fractures within each sample not visible to the naked eye, complementing conventional optical examination.

### Optical petrography

Polished sections were examined by optical microscopy under plane-polarized light (PPL) and cross-polarized light (XPL) condition, using a Zeiss AxioImager A2m polarizing microscope equipped with a bespoke Zeiss AxioCam ICC5 digital camera. This allowed identification of many of the minerals present in the rock samples and their textural relationships.

### Digital autoradiography

The distribution characteristics of total natural radioactivity in the rock matrix adjacent to main fractures was studied by digital autoradiography using storage phosphor imaging plate (IP) methodology. The IP acquires data relating to an ionising radiation source in two dimensions via an inorganic Eu-doped BaFBr phosphor layer, bonded to a polyester support material. When the IP is exposed to ionising radiation such as X-rays, gamma rays, alpha and beta particles, electron hole-pairs are created within the phosphor^39–41^ These hole-pairs are created in such a way that they store energy proportional to the amount of incident radiation^41^. This energy can be recovered via stimulation of the phosphor by a He-Ne laser, emitting red light at 635 nm, as photostimulated luminescence (PSL)^39,40^. This releases energy in the form of blue light emission from the phosphor in amounts proportional to the radioactivity that the phosphor was exposed to^41^. The IP technique cumulatively detects alpha-, beta- and gamma-radiation, as well as background cosmic radiation; total radioactivity is recorded and the different kinds of radioactivity are not discriminated.

The IPs were exposed to the samples for approximately 29 days (695 hours) in a light-tight dark box before removal under darkroom conditions. The IP was scanned with red laser light (635 nm) to produce photostimulated luminescence (PSL) images of the total distribution of radioactivity^39,40^.

### Etch track autoradiography

α- (etch track) autoradiographs show the distribution of α-emitting radionuclides very near to the surface of a sample. The method employed to obtain the autoradiographs followed closely the method described in^42^.

The autoradiographs were produced by placing a piece of allyl diglycol carbonate monomer (ADC) plastic detector medium in contact with the carefully cleaned surface of a thin section of rock. The ADC is left in contact with the rock surface for about 8 weeks. After being exposed to the rock in this way the ADC is then etched for 2–3 hours using 6 N NaOH solution. This process reveals tracks where the structure of the ADC has been damaged by α-particles. Each track is generated by an α-particle produced by a single nuclear decay.

### ICP-MS and ICP-OES analysis

Twelve 1 g samples were dissolved for analysis via plasma spectrometry following standard microwave assisted digestion in concentrated nitric/hydrofluoric acid and dilution in de-ionised water. In total, all samples were dissolved in 52 ml solution, 10 ml of which was then passed on for ICP analysis, 5 ml for ICP-AES and 5 ml for ICP-MS.

The inductively coupled plasma mass spectrometer (ICP-MS) used was an Agilent Technologies, UK model 7500×. This equipment was fitted with an autosampler (ASX-500 series), a quartz double-pass spray chamber (Peltier-cooled, Scott-type), a concentric MicroMist nebuliser and a 3rd generation Octopole Reaction System (ORS3). Optimisation of the ICP-MS was performed daily using a solution of Li, Mg, Y, Ce, Tl and Co (1 µg/L) in 2% (w/v) HNO_3_ acid (Agilent Technologies, UK). To correct for any matrix interferences or signal drift within each sample run, a 100 mg/L internal standard solution of 45Sc, 72Ge, 103Rh, 125Te and 193Ir was used.

Standards are made up from stock single and mixed element standards supplied by VWR and Johnson Matthey. Working standards are prepared by serial dilution, made up in sub-boiling point distilled HNO_3_ diluted to 2% (w/v) in 18.2 MΩ deionised water.

The ICP-OES analyses were undertaken using a Perkin-Elmer Optima 5300DV. The sample introduction system comprises a concentric glass nebulizer system fitted to a cyclonic spray chamber. This Optima 5000 DV spectrometer has the capability of viewing the plasma radially and axially. The spectrometer is based on an echelle polychromator with a segmented-array charge-coupled-device. The wavelength range is 163–782 nm. Standards and a blank are used for calibration and then run as unknowns. A batch of samples of 10 to 20 are run depending on the number of analytes required and then the blank and standards, this cycle is repeated. The standards are run at the end to complete the analysis. Analyzed elements in this case were: Ca, Mg, Na, K, Sr, Fe, Mn, Zn, Cu, and Ni.

Standards are made up from stock single and mixed element standards supplied by VWR and Johnson Matthey. Working standards are prepared by serial dilution, made up in sub-boiling point distilled HNO_3_ diluted to 2% in 18.2 MΩ deionised water.

### Powder X-ray diffraction

Standard XRD patterns of powders were produced by ball-milling fragments of rock sampled from the bulk and from the fracture infill areas. Analyses was completed using a Bruker D8 Advance diffractometer. Powder patterns were then interpreted using a standard search/match algorithm available within the manufacturer’s EVA software package. After a first “blind” attempt at fitting the patterns, a second round of search/match was completed using published information about the mineralogy of these rocks to refine the pattern fits and to take advantage of chemical information to constrain the search algorithm.

Measurements were completed using a parallel incident X-ray beam produce by a Goebel mirror attachment on the X-ray source tube. Scans were completed in standard θ/2θ geometry in locked coupled mode, with powders mounted on a low background Si wafer. Incident radiation was Cu K_α_ over a scan range of 5 to 70 degrees 2θ, 0.5 s/step, 0.02 degree step size. We used a LynxEye position sensitive detector and relied on quartz within the samples to act as an internal standard to verify angular position.

### Glancing incidence XRD

At low angle, the thin layer of surface precipitates dominates the XRD pattern, because the incident X-ray beam is optimized to interact with approximately the upper 100 μm of the surface, thereby minimizing the signal from the bulk rock. This method has previously proved successful with uranium-bearing micro-precipitates^43^.

Measurements were completed using flat-bottomed cut specimens. These measurements used a parallel incident X-ray beam in detector scanning mode, where the incident beam was held at constant angle relative to the fracture surface and the detector was scanned from twice the incident angle to 70 degrees 2θ, 0.2 s/step, 0.02 degree step size. Scans were completed with a range of incident angles from 4 to 16 degrees in steps of 2 degrees. This protocol allows patterns at different angles to be compared and may allow depth profiles to be resolved: as the incident angle increases the incident beam penetrates further into the surface and therefore phases which decrease in intensity as a function of incident angle indicate phases that are concentrated at the surface. In addition, changes in peak intensity as a function of angle may give information concerning crystal orientation or phase surface coverage. Incident radiation was Cu Kα. A scintillation counter was used with a quartz standard analysed independently to calibrate peak positions.

### EDXA X-ray element mapping

EDXA element maps were recorded using the ‘Mapping’ programme within the Oxford Energy INCA Suite 5.04 Issue 21a + SP2 (2012) software package on a FEI QUANTA 600 scanning electron microscope. The samples were uncoated, and the mapping was undertaken in low vacuum mode (0.98 torr). EDXA X-ray spectral data were recorded from each pixel within the field of view at a minimum resolution of 512 ×512 pixels, using a 20 kV electron beam, ~0.45 nA beam current and at a working distance of 10 mm, to give optimum X-ray count rates of between 6,500 and 10,000 counts per second. X-ray element maps were produced by summation of data recorded from multiple frame scans to produce maps with sufficient X-ray counts per pixel to enable key elements, required for the differentiation of the mineral species present, to be detected above background noise. Maps were recorded and summed over 30 frame scans to achieve sufficient analytical sensitivity within a practical time limit of around 1 hour per mapped area.

### BSEM investigation of microstructures

The polished sections prepared following vacuum-impregnation with blue-dyed epoxy-resin were examined by optical microscopy under plane-polarized light (PPL) and cross-polarized light (XPL) condition, using a Zeiss AxioImager A2m polarizing microscope equipped with a bespoke Zeiss AxioCam ICC5 digital camera. They were also examined by backscattered scanning electron microscopy (BSEM) using a FEI QUANTA 600 ESEM.

The thin sections were qualitatively assessed over their entire area by BSEM and optical microscopy observation. Quantitative fracture intensity measurements were made largely from BSEM images recorded along a linear transect drawn perpendicular to the trace of the PFF in the proximal thin section (A, Fig. 2). A continuous sequence of adjacent fields of view were observed across the thin section along this transect. Each field of view was between 0.3 and 1 mm wide, depending on the magnification required to image and resolve the features. The fracture intensity was determined by counting the intersection of microfracture traces along the central horizontal line within each field of view observed. Only microfractures which were obviously open or porous (as seen in thin section) were included in the counting statistics. The Toki granite samples contained numerous healed microfractures, hosted particularly in quartz crystals. These were obvious from fine inclusion trails but were non-porous and consequently, these were excluded from the microfracture counts. The fracture frequency was simply estimated as follows:

For comparison, an indication of “background” fracture intensity was determined from fracture counting in representative fields of view observed from the “distal” polished thin section (B, Fig. 2). The distance of these measurement points was determined by extrapolation to the polished section position along a line perpendicular to the plane of the main fracture.

The approach described above introduces a sampling bias in the counting analysis. The microfractures recorded will be strongly biased towards fractures that intersect the transect at a high angle (i.e. fractures parallel or sub-parallel to the main fracture). Fractures that have an orientation parallel, or sub-parallel, to the transect will not be sampled or will be under-represented.

Three categories of microfracture types were defined:

- Grain boundary microfractures:
  Microfractures around the margins of discrete grains or crystals, and developed between adjacent grains or crystals of similar or different mineralogy. Their form is defined by the external morphology of mineral grains or crystals. These microfractures may interconnect with “intra-granular” (“intra-crystalline”) microfractures that reach the grain or crystal boundary. They may also connect with “trans-granular” (“trans-crystalline”) microfractures that cut across the rock fabric;
- Intra-granular (or intra-crystalline) microfractures:
  Microfractures developed and confined within the margins of discrete grains or crystals. These may form regularly organised networks within mineral grains (e.g. developed by dilation along crystal cleavages) or irregularly “non-systematic”. They may form interconnected networks with “grain boundary” microfractures where they reach the grain boundaries, or may connect with cross-cutting “trans-granular” microfractures. However, they may also occur as isolated features or networks within mineral grains that may not necessarily connect with the larger external microfracture network. Some previous studies have differentiated cleavage microfractures as a separate category^11^, however, cleavage microfractures are included here within a general intra-crystalline microfracture category;
- Trans-granular (or trans-crystalline) microfractures:

 Microfractures which cross grain boundaries between adjacent mineral grains. They generally have longer trace lengths that “grain boundary” or “intra-granular” microfractures. They can intersect both these types of microfractures to provide a more extensive connected microfracture network.

### EMPA

EMPA analysis was completed using a Cameca SX-100 Electron Microprobe housed within the Williamson Research Centre for Molecular Environmental Science at the School of Earth and Environmental Science, University of Manchester. The probe is fitted with five wavelength dispersive spectrometers for quantitative analysis.

Maps were completed using an incident electron beam of 30 kV energy, 100 nA current, dwell time of 0.02 seconds per pixel, beam spot size approximately 2 μm diameter. WDS spectrometers were optimized for peak positions of the elements of interest, and then the specimen was rastered through the beam with characteristic intensities for each element recorded as a function of specimen position. In this way, five elements can be mapped at a time. Back scattered electron images were also recorded. Full WDS scans at single points which showed high trace element concentrations were also acquired in order to confirm that the intensity maps were actually characteristic emission events and not fluctuations in background or peak shouldering. Elements mapped include: Na, Mg, Al, Si, Ca, K, Ti, Fe, Mn, for all maps. Additional elements in some regions were: Cl, S, Ni, Sr, Ba, Cr, Th, Y, U.

Linescans were acquired by defining two end points of a traverse across an area of interest and then programming a given number of points to be acquired within that linear distance. Point spacing varied between 70 and 10 microns, depending on the specimen. Incident electron beam energy was 15 kV, 4 nA current, beam spot size approximately 2 μA current, beam spot analysed were: Na, Mg, Al, Si, P, Ca, K, Ti, Fe, Mn, Cl, S, Ni, Cr, Th, Y, U.

### Synchrotron analyses

Measurements were completed at the Diamond Light Source beamline I-18, which is an undulator beamline optimized for microfocus mapping and X-ray absorption spectroscopy. Beam spot size is controlled by a pair of Kirkpatrick-Baez focusing mirrors and incident beam energy was set by a double crystal monochromator using a pair of Si(111) crystals. Detection of fluoresced characteristic X-rays is via a four element Si-drifted solid-state Vortex detector set at 90° scattering angle to the incident beam with motor control so that detector-sample distance can be changed depending on emitted intensities. The beamline also includes several attenuators, which can be placed into the incident beam path in order to cut incident intensity in the case where fluorescence overwhelms the data collection rate of the detection system. Samples are mounted on a motorized stage which can be rastered for mapping. Software records map positions and fluorescence intensities as a function of emitted energy at each point. The energy of the channels in the Vortex energy dispersive detector was calibrated by measuring the position of the K absorption edge of a pure metal Y standard. The theoretical value is 17038 eV. In our experiments, the detector gave this value as 17036.4 eV, and therefore a + 1.6 eV offset was applied to the spectra obtained for the granite specimens. For this study, we compare the edge energies in our unknowns with well-constrained edge determinations for mineral specimens as previously determined^35^. In that study, the U(IV) edge was determined to be 17166.6 eV in a doped anorthite glass, and the U(VI) edge was determined to be at 17169.4 eV. Edge energies here were determined assuming the edge is located at ½ μ on the normalized absorption spectrum^35^. Thus, the edge energy difference here between fully oxidized and fully reduced uranium should be approximately 2.8 eV. Due to the low concentrations of U, the appearance of a monochromator glitch at 17192 eV, and interference from the Rb Kα emission, the U spectra are relatively noisy (red curve especially). The combination of these analytical problems with time constraints meant we were unable to acquire EXAFS for U within the fracture infill. Such data would assist in unambiguously determining the secondary phase which has incorporated this oxidized and mobilized actinide. Durango apatite was used as a mineral standard for point analysis^44^. Data were processed using Athena^45^ and PyMCA^46^.

### X-ray computed tomography (μ-CT)

All specimens were analyzed using a Nikon Metris 225/320 kV CT system housed in a customized bay at the Henry Moseley X-Ray Imaging Facility (HMXIF) which has the following benefits:

Fast analysis times can be used to minimize blurring during *in-situ* experiments.

The superior detector (2 K x 2 K Perkin Elmer 1621–16-bit amorphous silicon flat-panel detector with 200 µm pixel pitch) allows finer differences in contrast to be detected. Many of the features observed in this experiment would not have been possible on a standard Nikon 225 kV X-ray CT instrument.

The increased detector size allows the specimen to be viewed closer to the source thus improving image resolution.

The ability to change the target material allows optimising the photon count for a specific energy range. In this case a tungsten target produced the best signal to noise ratio for the accelerating voltage of 100 kV and a current of 44 mA.

Specimens were mounted onto the Nikon CT system rotation stage. The specimen to source distance was 37 mm and the source to detector distance was 970 mm. During the XCT analysis the specimen was rotated over a 360° rotation range for a fixed time period collecting 4001 projections per scan with a voxel size of 3.7 µm x 3.7 µm x 3.7 µm. Focus was optimized for the low energy spectrum and the system alignment was rechecked in order to optimize resolution.

After image acquisition, data sets were filtered and reconstructed using the Nikon Metris CT-Pro reconstruction software. Filtering enhances the grey-scale contrast, while reconstruction renders the individual scans into 3-D volumes. The combined data set was also resampled in order to make it feasible to handle during processing. After these processing steps a working voxel size of 11.5 µm x 11.5 µm x 11.5 µm was obtained. Segmented phase densities and color assignments are given in Table S7. Islanding was used in image analysis such that features with length scales smaller than 100 μIslanding was us

A high-resolution scan of MIU-3/10 was also completed using a ZEISS Xradia Versa 520 ×-ray microscope using a 4X optical lens, also part of the HMXIF. The Zeiss Xradia Versa 520 is a sub-micron resolution instrument with extensive imaging capabilities. The system bridges the gap in resolution between the traditional lower resolution (~100 to 5 μm) geometric magnification systems and the high resolution (~20 nm to 200 nm) X-ray optical systems. For the MIU-3/10 small core an accelerating voltage of 80 kV at 10 watts power was used. During the XCT analysis the specimen was rotated over a 360° rotation range for a fixed time period collecting 1601 projections per scan with a voxel size of 1.77 µm x 1.77 µm x 1.77 µm. After filtering, the data set was reconstructed and resampled to produce a working voxel size of 5.31 µm x 5.31 µm x 5.31 µm. For this scan, islanding excluded features with a length scale smaller than 50 μFo

### Gamma spectrometry

Powders of samples taken from bulk offcut material distal to the main fracture feature and similar powders prepared from offcut shards directly within the altered fracture infill were used. These powders are the same as those used in XRD analysis of these specimens. The measurement protocol was as follows:

~1.2 g was extracted from a homogenized fine grain size powder.

Each specimen was sealed in a Nalgene bottle with epoxy resin for a minimum of 27 days. This period allowed secular equilibrium between ^226^Ra and its daughter ^214^Bi to be established.

Gamma ray emissions as a function of energy were then detected and counted for 24 hours using a high precision germanium detector system. The most sensitive diagnostic peak for radium is at 609 keV. This is for ^214^Bi, which is part of the ^226^Ra decay chain. Background counts were measured in separate measurements in order to calibrate the detection and allow conversion of raw counts to Bq kg^−1^.

## Supplementary information

Supplementary Information.

Video of CT scan of sample MIU 3-10.

## Data Availability

The data that support the findings of this study are available from the corresponding author upon reasonable request. The source data files for all figures and tables are listed in a source data file.
