# Supplementary material for: Mineral reaction kinetics constrain the length scale of rock matrix diffusion
Source: Sci Rep. 2020 May 18;10:8142. doi: 10.1038/s41598-020-65113-x (PMC7235024; doi:10.1038/s41598-020-65113-x)
Supplement: Supplementary file 1 — Supplementary Information. [file 41598_2020_65113_MOESM1_ESM.pdf]

Supplementary Information

Mineral reaction kinetics constrain the length scale of rock matrix diffusion

Wogelius R.A.*[1], Milodowski A.E.[2], Field L.P.[2], Metcalfe R.[3], Lowe T.[4], van Veelen A.[5], Carpenter G.[6], Norris S.[7], Yardley B.[8]

[1]University of Manchester, School of Earth and Environmental Sciences, Williamson Research Centre for Molecular Environmental Science & Interdisciplinary Centre for Ancient Life, Manchester, M13 9PL, UK
[2]British Geological Survey, British Geological Survey, Environmental Science Centre, Nicker Hill, Keyworth, Nottingham, NG12 5GG, UK
[3]Quintessa Limited, The Hub, 14 Station Road, Henley-on-Thames, Oxfordshire, RG9 1AY, UK
[4] Henry Moseley X-ray Imaging Facility, The University of Manchester, Upper Brook Street, Manchester, M13 9PY, UK
[5]University of Southampton, Bioengineering Sciences Research Group, Southampton, SO17 1B, UK
[6]NSG Environmental Ltd, Festival House, Jessop Avenue, Cheltenham, Gloucestershire, GL50 3SH, UK
[7]Radioactive Waste Management Limited, Building 587, Curie Avenue, Harwell Science and Innovation Campus, Didcot, Oxfordshire, OX11 0RH, UK
[8]School of Earth and Environment, University of Leeds, Leeds, LS2 9JT, UK

*corresponding author

Supplementary Note 1. Toki region geology and sample selection criteria

The granite is overlain unconformably by the Miocene Mizunami Group (20–15 Ma) and the Pliocene Seto Group (5–0.7 Ma). Both the Toki Granite and the Mizunami Group are displaced by the approximately E–W-striking Tsukiyoshi Fault, which dips to the south at c.60° to c.70° and has a reverse displacement of c.30 m[43]. Further background geological information relevant to Mizunami is available[43-47].

The rock samples studied here were taken from rock core obtained from the MIU-3 borehole, which was drilled into the Toki Granite by JNC at the Shobasama site in Mizunami, central Japan. The key objective in selecting the samples was to investigate rock matrix diffusion processes in the wallrock adjacent to fractures that could be considered to be potentially hydraulically-active and closely associated with the present-day low-temperature groundwater flow system (as opposed to geologically-old hydrothermal activity). Identification of potentially flowing fracture features was based on detailed fracture logging of the distribution of the youngest fracture mineralisation recognised (from prior petrological studies) and its comparison with the distribution of groundwater inflows identified from geophysical and hydrogeological borehole testing. This approach has been successfully used to identify potential flowing features in previous palaeohydrogeological studies[26 and references therein], and used a combination of the following principal criteria:

- Presence of open, intact fractures with undisturbed "gapped" aperture;
- Presence vuggy porosity lined by euhedral late-stage calcite crystals, indicative of crystal growth into open pore space;
- Evidence of mineral dissolution and secondary porosity in the fracture mineralisation and / or rock matrix;
- Presence of the youngest generation of fracture mineralisation: late-stage calcite, often associated with pyrite and smectitic clay in the Toki Granite; late-stage calcite and laumontite (which were also found as suspended material in groundwater during pumping tests) in the Carnmenellis Granite;
- Correspondence with location of points or zones of groundwater inflow in the boreholes detected from hydrogeological and / or wireline geophysical testing.

Supplementary Note 2. Solid phase volume change calculation

3 anorthite + 2 phlogopite + 3 HCO_3^- + 5H+ -> 3 calcite + clinochlore + 3 quartz + 2 muscovite + Mg^{+2}

3*103.12 + 2*149.66 = 608.68

3*36.90 + 207.11 + 3*22.69 + 2*143.6 = 673.08

64.4 cm^3 positive delta V, 10% volume increase.

Molar volumes taken from compiled values[48].

Supplementary Note 3. Explanation of Supplementary Video 1.

This is a video presenting the lower-resolution CT scan of core sampled from MIU-3/10, comparable to Supplementary Figure 1A-G. Initial voxel size was 3.7 µm x 3.7 µm x 3.7 µm. Data has been filtered, reconstructed, and resampled to produce a working voxel size of 11.5 µm x 11.5 µm x 11.5 µm. Segmented phase densities and color assignments are given in Table S7. Islanding was used in image analysis such that features with length scales smaller than 100 µm were excluded. Raw data (black and white) transforms to the segmented phases. Finally, at the end, calcite is added (purple) to the high density oxides (gold) and pores (blue). The calcite is made semi-transparent to show it is ubiquitous but not monolithic. This shows that secondary precipitates (calcite in this case) can drastically fill pores and decrease fluid access to the bulk core volume.

Supplementary References

43. Hama K, Amano K, Metcalfe R, Yoshida H, Iwatsuki T, Milodowski AE, & Gillespie MR, Mineralogical and petrological evidence for the hydrogeological characteristics of the Tsukiyoshi Fault, Japan. Quarterly Journal of Engineering Geology and Hydrogeology, 35, 189–202, 2002

44. Wakita K, Melanges of the Mino terrane. Geological Society of Japan Memoir, 55,145–163 (in Japanese with English abstract), 2000.

45. Kawada K, Yamada N, Isomi H, Murayama M, & Katada M, Geology of Japanese Central Alps and its western area (2) The Nohi rhyolites. Chikyu-Kagaku, 54, 20–31. (in Japanese with English abstract), 1961.

46. Yamashita N, Kasuno Y & Itoigawa J, Japanese geology, 5. Chubu district II. Kyoritsu shuppan, Tokyo (in Japanese), 1988.

47. Nishimoto S & Yoshida H, Hydrothermal alteration of deep fractured granite: effect of dissolution and precipitation. Lithos, 115:153–162, 2010.

48. http://database.iem.ac.ru/mincryst/

49. Bethke C. "Geochemical and Biogeochemical Reaction Modelling", 564 p., Cambridge Press, 2008.

50. Deer WA, Howie RA & Zussman J, An Introduction to the Rock-Forming Minerals. (Second Edition). Pearson / Prentice-Hall, Harlow, England, 696pp, 1992.

51. Chaboy J., & Díaz-Moreno S. (2011) "Ab Initio X-ray Absorption Spectroscopy Study of the Solvation Structure of Th(IV), U(IV), and Np(IV) in Aqueous Solution," *Journal of Physical Chemistry A* **115**, 2345-2349.

Supplementary Table 1. Electron Microprobe Linescans with compositions of host phases.

Specimen	Location	Total	Th. Total	Mineral	Phase type
Toki, less altered	MIU_3/8_Area1_Line1	100.54	100	Plagioclase Ab71	Primary
Toki, less altered	MIU_3/8_Area2_Line1	94.89	96	Biotite	Primary
Toki, less altered	MIU_3/8_Area2_Line1	95.01	96	Apatite	La/Y/Ac host
Toki, less altered	MIU_3/8_Area2_Line2	100.46	100	Plagioclase Ab92	Primary
Toki, less altered	MIU_3/8_Area3_Line1	100.65	100	Plagioclase Ab89	Primary
Toki, less altered	MIU_3/8_Area3_Line2	99.38	100	K-feldspar Or90	Primary
Toki, more altered	MIU_3/10_Area1_Line1	98.97	100	Plagioclase Ab93	Primary
Toki, more altered	MIU_3/10_Area1_Line2	101.07	100	Plagioclase Ab92	Primary
Toki, more altered	MIU_3/10_Area1_Line2	100.6	100	K-feldspar Or97	Primary
Toki, more altered	MIU_3/10_Area1_Line2	86.67	88	Clinochlore	Secondary
Toki, more altered	MIU_3/10_Area2_Line1	100.95	100	Plagioclase Ab93	Primary
Toki, more altered	MIU_3/10_Area3_Line1	87.89	88	Clinochlore	Secondary
Toki, more altered	MIU_3/10_Area4_Line1	99.55	100	Plagioclase Ab94	Primary

Supplementary Table 2 Groundwater Analyses. Water samples from immediately above (MIU-3-2) and immediately below (MIU-3-3) the Toki Granite samples MIU-3/10 (522 m depth) and MIU-3/8 (555 m depth).

**Locality**		**Shobasama, Mizunami, Japan**	**Shobasama, Mizunami, Japan**
**Borehole**		**MIU-3**	**MIU-3**
**Water sample**		MIU-3-2	MIU-3-3
**Water sample depth (mbgl)**	mbgl	240.48-319.28	604.88-690.78
**Source**		3	3
**Temperature**	**˚C**	21	9.9
**pH**		9.31	9.24
**TDS**	mg/L	N.R.	N.R.
**Na**	mg/L	42	53
**K**	mg/L	0.88	0.71
**Ca**	mg/L	2.5	3.6
**Mg**	mg/L	0.21	0.14
**FeTotal**	mg/L	N.R.	N.R.
**Si**	mg/L	7.2	8.1
**Sr**	mg/L	0.022	0.052
**Cl**	mg/L	20	51
**SO4**	mg/L	1.3	<0.2
**HCO3**	mg/L	98	73
**TIC**	mg/L	18	14
**Br**	mg/L	0.0053	0.0061
**B**	mg/L	0.23	0.14
**U**	mg/L	N.R.	N.R.
**Th**	mg/L	N.R.	N.R.
		Log Q/K	Log Q/K
**Antigorite**		7.44	usatd
**Talc**		**2.19**	**0.52**
**Strontianite**		**0.80**	**0.98**
**Dolomite**		0.67	usatd
**Dolomite-ord**		0.67	usatd
**Tremolite**		0.65	usatd
**Calcite**		**0.31**	**0.14**
**Aragonite**		0.14	usatd
**Chrysotile**		0.12	usatd
**Quartz**		**0.04**	**0.34**

Note: N.R. = Not Reported

Log Q/K is $\log_{10}$ of reaction quotient divided by equilibrium constant calculated by Geochemist's Workbench version 11[49]. Positive values indicate supersaturation; usatd indicates the phase is undersaturated at these conditions.

Supplementary Table 3. CT pore volume fractions

		Pore volume fraction	Calcite volume fraction
Toki	MIU-3/10	6.0×10^{-4}	0.192
Toki	MIU-3/8	1.1×10^{-3}	
BVG	70953	2.4×10^{-5}	
BVG	70955	5.0×10^{-6}	
Rosemanowes	70951	5.4×10^{-4}	
Toki, high res.	MIU-3/10	8.4×10^{-3}	

Supplementary Table 4. Summary of XRD results. Grey filled boxes indicate minerals interpreted to be secondary phases. X denotes presence, and multiple x's indicate qualitative abundance based on peak intensities.

			Qtz	Plag.	K-spar	Biotite	Clinochlore	Calcite	Sericite (Musc)	Hematite	TiO_2*
Toki Granite	Well developed	Bulk	x	x	x		x				
	"3/10"	Infill	xx	x	x		x	**xxx**	x		
	Less developed	Bulk	x	x	x	x	x				
	"3/8"	Infill	xx	x	x		x				
Carnmenellis Granite		Bulk	x	x	x		x	x	x		
		Infill	x	x	x			?			x

Clinochlore: $(Mg,Fe)_5Al(AlSi_3O_{10})(OH)_8$

Sericite (musc): $KAl_2(AlSi_3O_{10})(OH)_2$

K-Feldspar: $KAlSi_3O_8$

Hematite: Fe_2O_3

Biotite: $K(Mg,Fe)_3[AlSi_3O_{10}(OH,F)_2$

Plagioclase: $NaAlSi_3O_8 – CaAl_2Si_2O_8$

Calcite: $CaCO_3$

Quartz: SiO_2

Supplementary Table 5. A) ICP-OES Data

	Toki, less dev.		Toki, more dev.	
ICP-OES	MIU3/8	MIU3/8	MIU3/10	MIU3/10
(ppm or %)	bulk	fracture	bulk	fracture
Ca	1.50%	0.50%	1.15%	31.40%
Mg	0.21%	699	986	323
Na	4.21%	2.09%	2.90%	745
K	3.57%	3.12%	2.79%	0.13%
Sr	128	61	107	79
Fe	1.89%	0.84%	1.06%	841
Mn	1036	303	607	0.19%
Zn	35	n.d.	1	n.d.
Cu	n.d.	n.d.	n.d.	n.d.
Ni	56	n.d.	n.d.	n.d.

Supplementary Table 5. B) ICP-MS Data

	Toki, less dev.		Toki, more dev.	
	MIU3/8	MIU3/8	MIU3/10	MIU3/10
ICP-MS (ppm)	bulk	fracture	bulk	fracture
Sr	99.43325	39.66617	80.70612	43.4908
Y	72.79198	36.94271	40.57775	185.9965
La	43.30116	16.07592	26.55986	9.25429
Ce	93.18072	36.49329	51.90435	21.59964
Pr	9.872615	3.909937	5.397682	2.785046
Nd	40.06154	15.77301	20.70921	14.06108
Sm	9.088837	3.845184	4.574378	5.015417
Eu	0.70032	0.366506	0.539652	0.675966
Gd	9.199812	4.122416	4.900333	8.342689
Tb	1.607175	0.764118	0.831405	1.683657
Dy	11.10383	5.480716	5.747061	13.87327
Ho	2.36809	1.18635	1.248057	3.63094
Er	7.673964	3.889745	4.015081	11.95577
Tm	1.267525	0.662354	0.637977	1.610513
Yb	9.169383	4.856519	4.367854	8.806947
Lu	1.374549	0.725216	0.66824	1.225752
Th	182.6449	71.48466	33.34202	1.833062
U	12.46682	6.011805	15.26164	12.71476

Supplementary Table 6. Qualitative XRF point analyses (Units are ppm except % where noted).

	MIU-3/8 Point Analyses				
	Bastnaesite	AcO_2 pt 1	AcO_2 pt 2	AcO_2 pt 3	Zircon
Ca	5.06%				
Th	3.08%	55.86%	62.62%	52.21%	1.93%
U	24	7434	1785	6.83%	4.86%
La	7.45%	5.34%	4.13%	1455	6054
Ce	18.47%	7.20%	10.64%	850	733
Y	4406	24.73%	12.50%	2.15%	17.56%
Nd		2.34%	3.35%		
Zr					20.32%
Hf					8.27%
Σ (cation) meas.	34.06%	95.47%	93.24%	61.19%	52.94%
Σ (cation) theor.	64%	88%	88%	88%	50%

Bastnaesite: large crystal in map scan 85535
AcO2 pt 1 and 2: Very small high Th+Y grains in map scan 85535
AcO2 pt 3: Small Th+U grain in map scan 85591
Zircon: Associated with biotite in map scan 85596

Supplementary Table 7. Phase densities[50] and segment assignments for CT images

**Mineral**	**Mass density (g/cm^3)**	**Segment**	**Scan Colour**
Thorianite	9.7	Highest	Yellow
Hematite	5.28	Highest	
Bastnaesite	5.02	Highest	
Zircon	4.85	Highest	
Goethite	3,3	Second	Green or Red
Apatite	3.17	Second	
Biotite	3.09	Second	
Clinochlore	2.83	Second	
Muscovite/Sericite	2.83	Second	
Calcite	2.71	Third	Purple
Plagioclase	2.63 Ab_{95} (2.76-2.61)	Fourth	Light Blue
Quartz	2.65	Fourth	
Microcline	2.56	Fourth	
Smectite	2.35 (2-2.7)	Fourth	
Pore	<<1	Lowest	Dark Blue

Supplementary Figure 1. CT Scans of MIU-3/10 and MIU-3/8.

A.

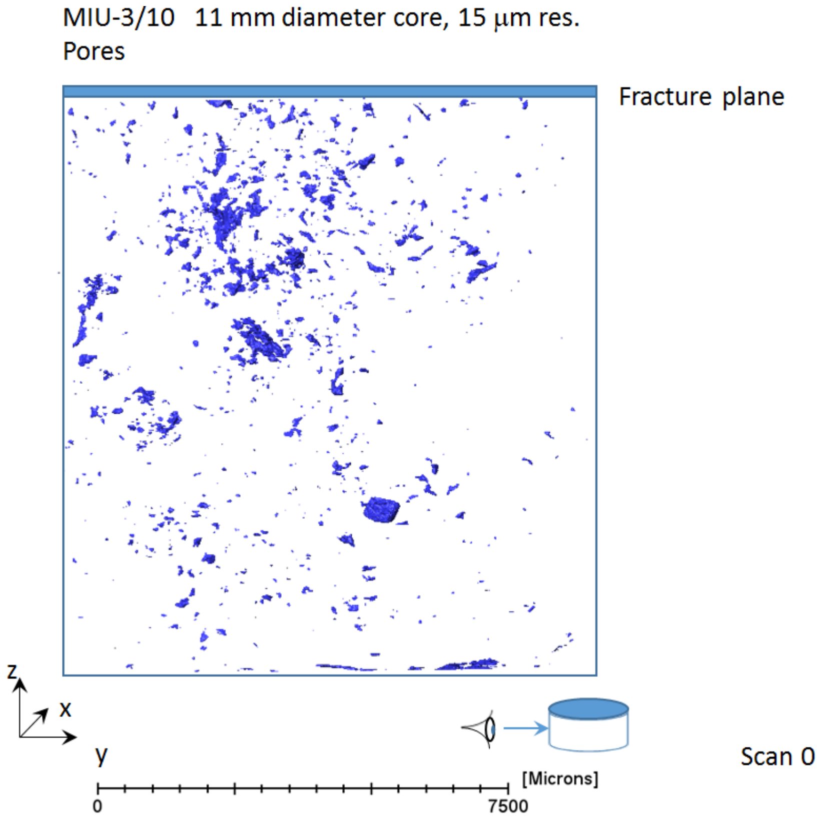

B.

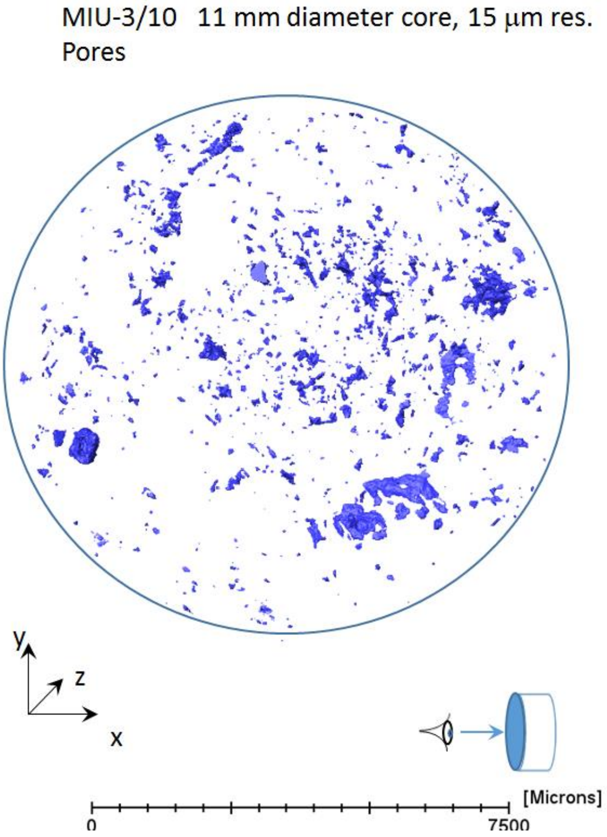

C.

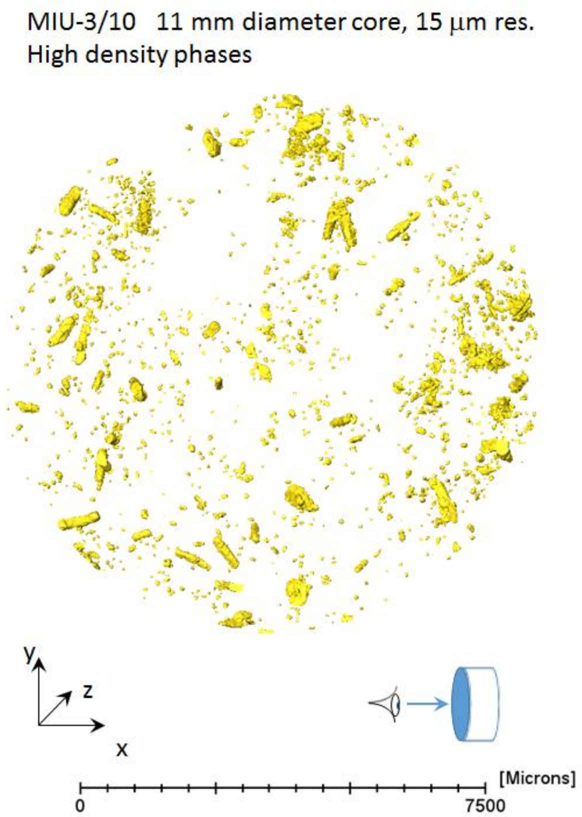

MIU-3/10 11 mm diameter core, 15 μm res.
High density phases
y
z
x
[Microns]
0
7500

D.

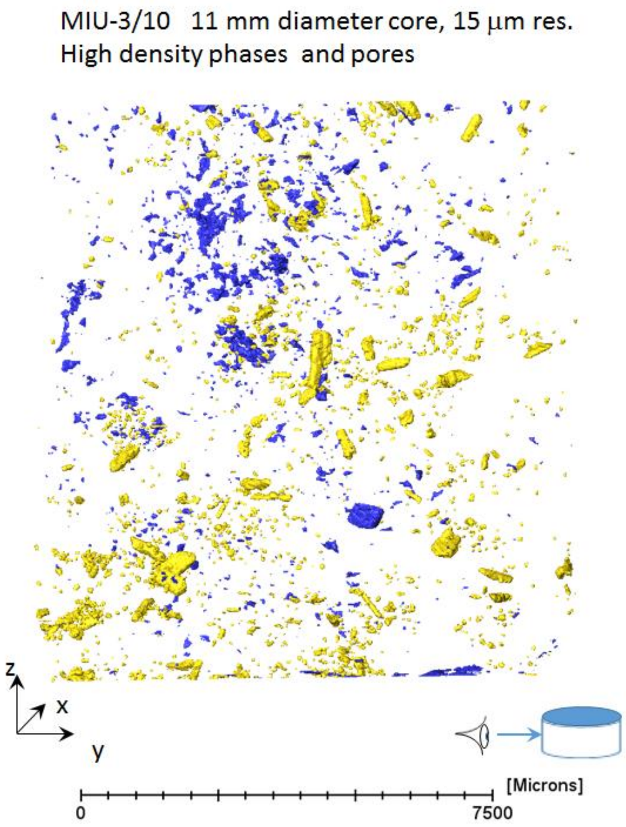

MIU-3/10 11 mm diameter core, 15 μm res.
High density phases and pores
z
x
y
[Microns]
0
7500

E.

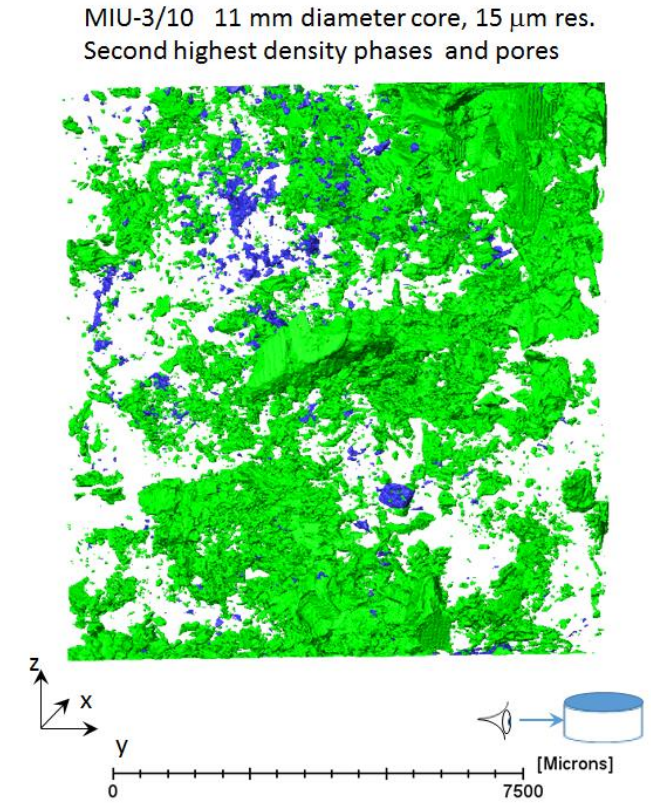
MIU-3/10 11 mm diameter core, 15 μm res.
Second highest density phases and pores
z
x
y
[Microns]
0
7500

F.

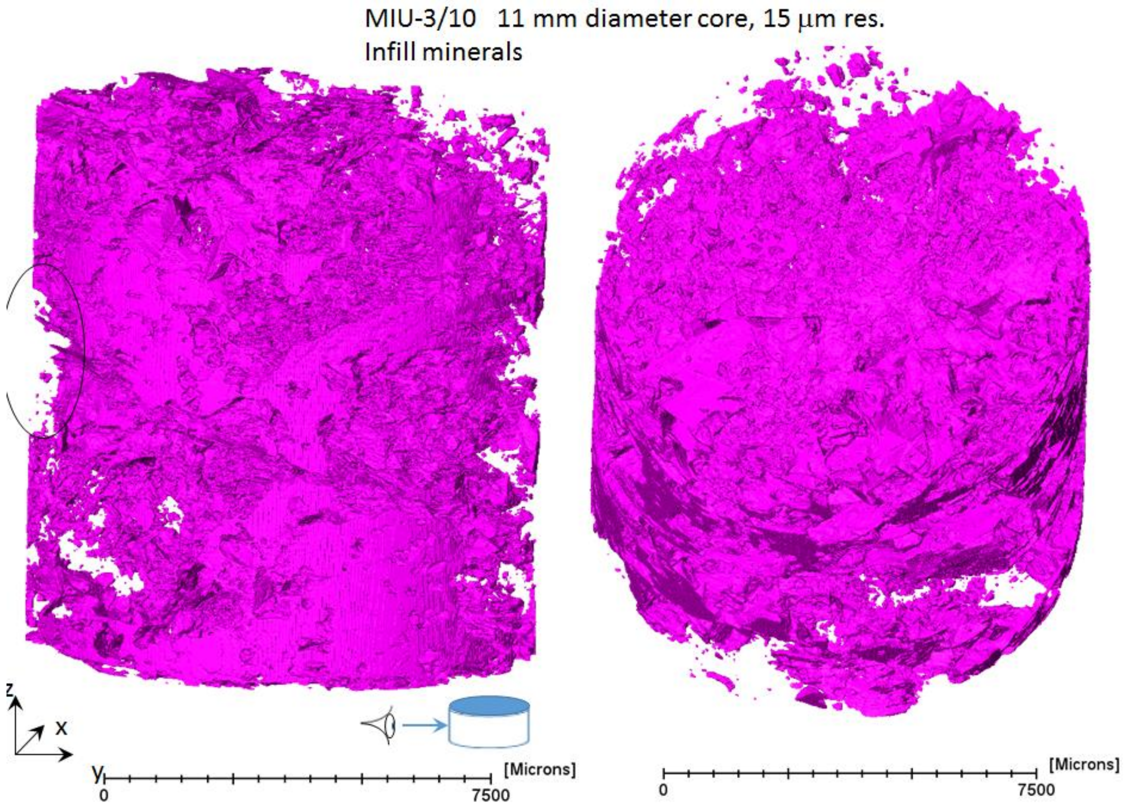
MIU-3/10 11 mm diameter core, 15 μm res.
Infill minerals
z
x
y
[Microns]
0
7500
[Microns]
0
7500

G.

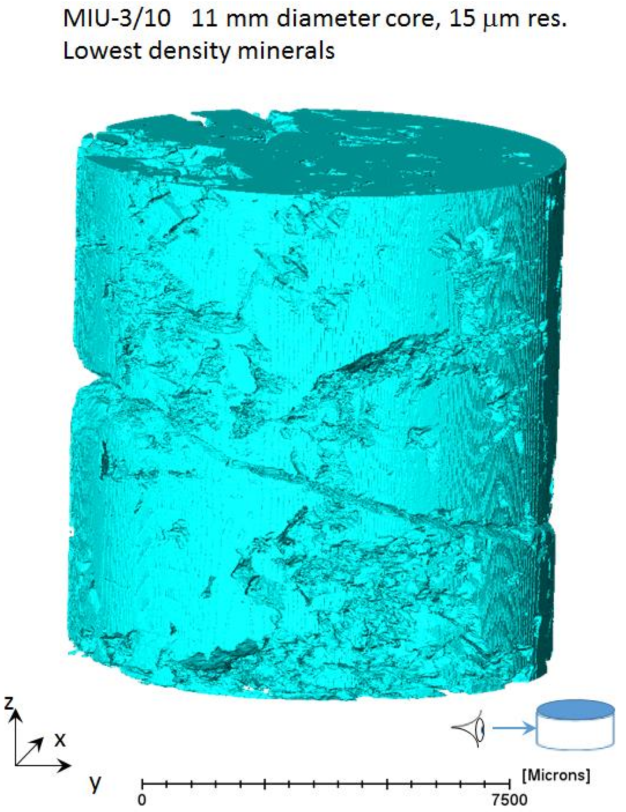
MIU-3/10 11 mm diameter core, 15 µm res.
Lowest density minerals
z
x
y
[Microns]
0
7500

H.

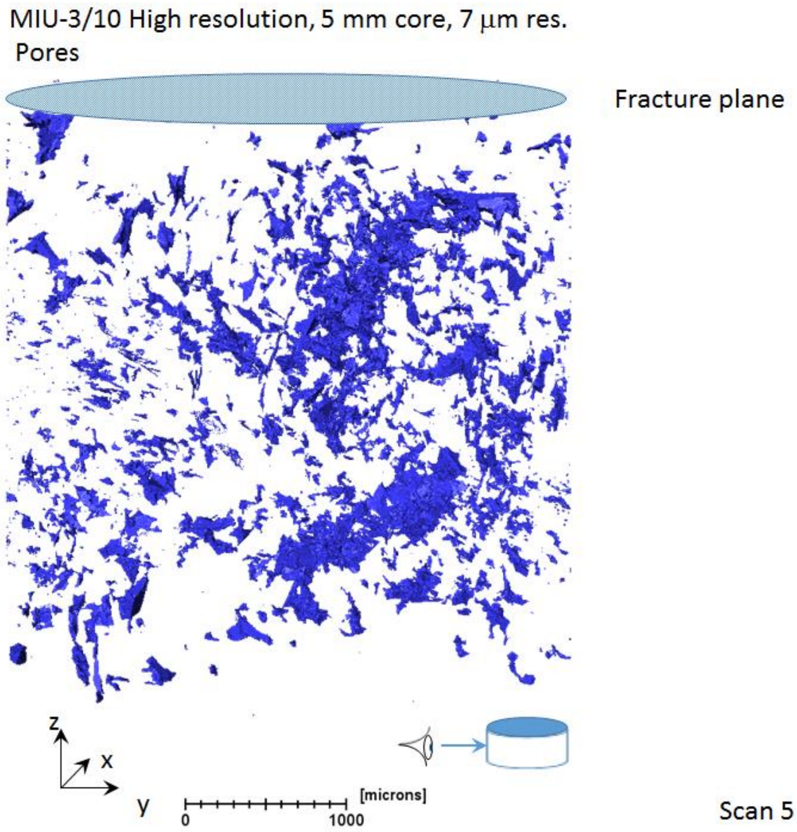
MIU-3/10 High resolution, 5 mm core, 7 µm res.
Pores
Fracture plane
z
x
y
[microns]
0
1000
Scan 5

I.

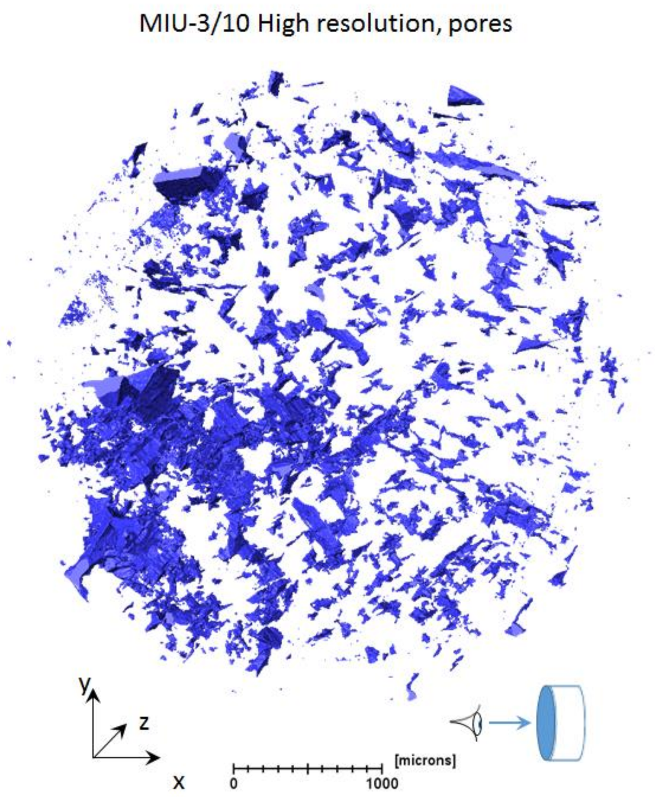
MIU-3/10 High resolution, pores
y
z
x
[microns]
0
1000

J.

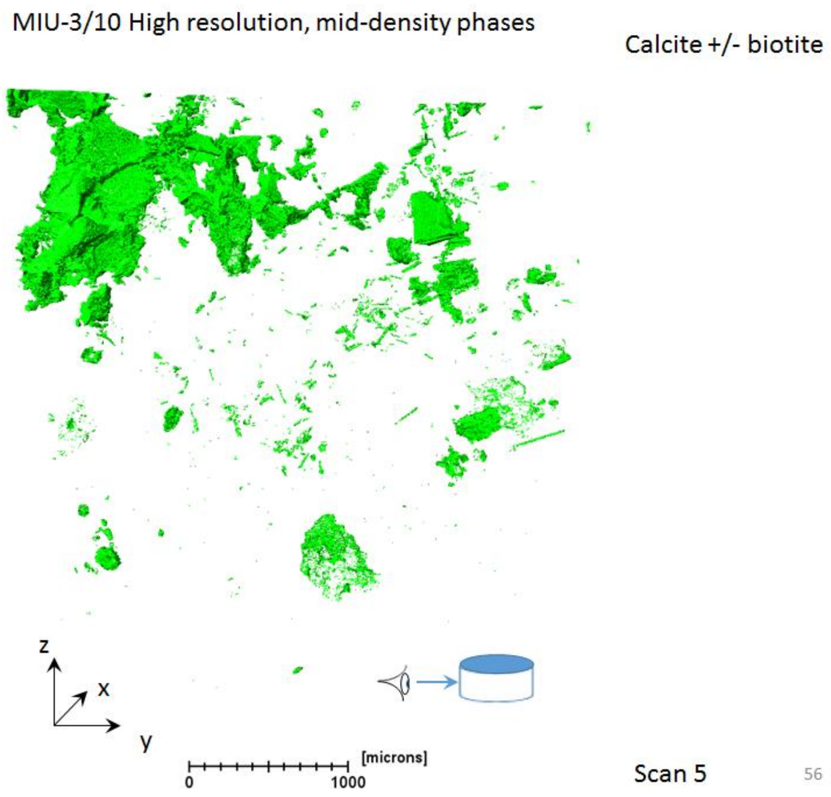
MIU-3/10 High resolution, mid-density phases
Calcite +/- biotite
z
x
y
[microns]
0
1000
Scan 5
56

K.

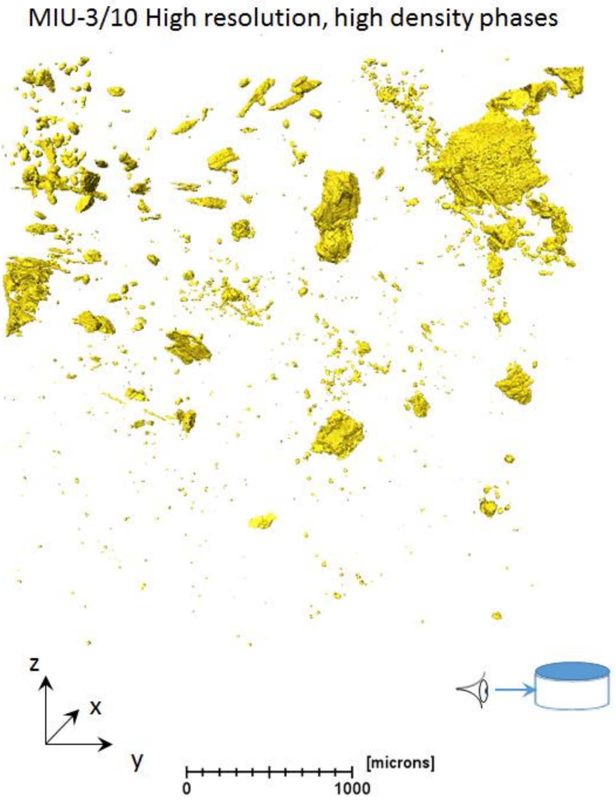
MIU-3/10 High resolution, high density phases
z
x
y
[microns]
0
1000

L.

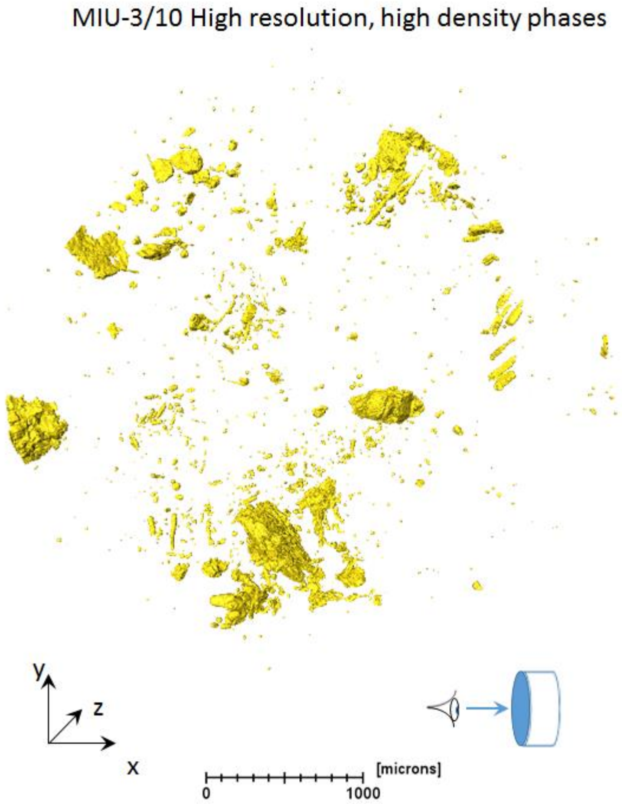
MIU-3/10 High resolution, high density phases
y
z
x
[microns]
0
1000

M.

MIU-3/10 high resolution, mid and high density phases

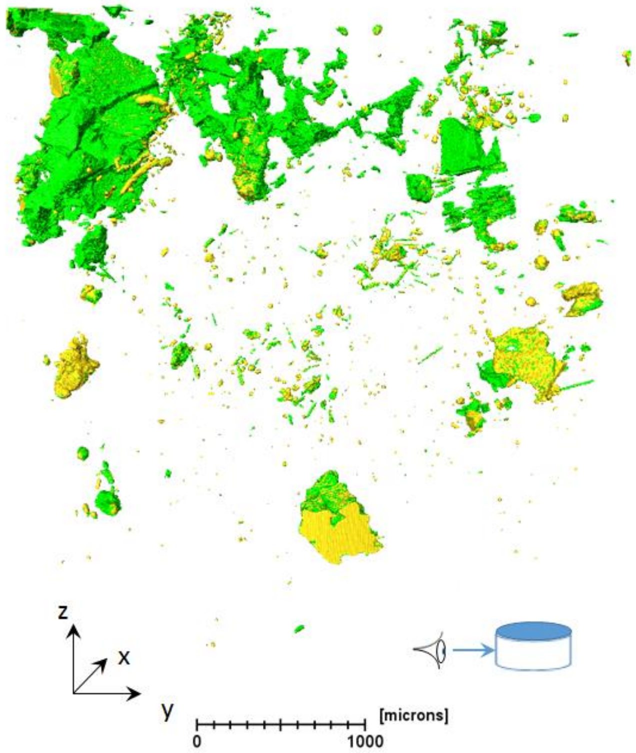

S

N.

MIU-3/10 High resolution, high density phases plus pores

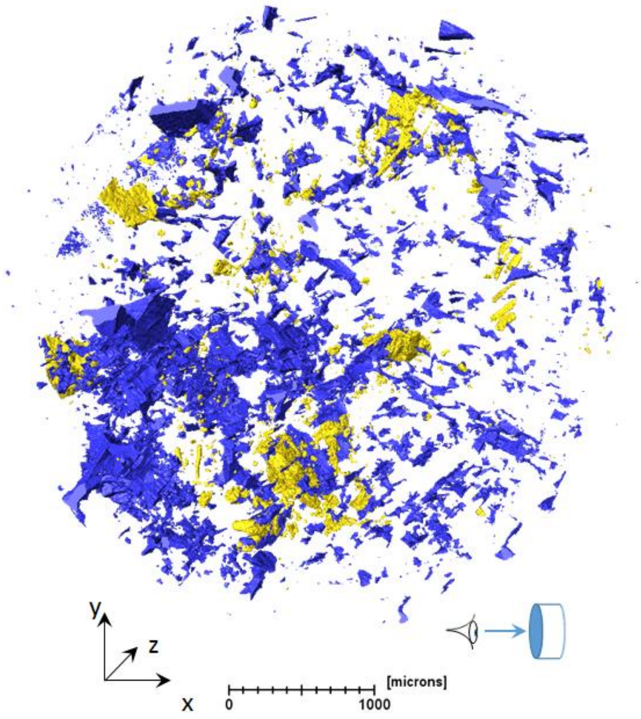

Scan 5

O.

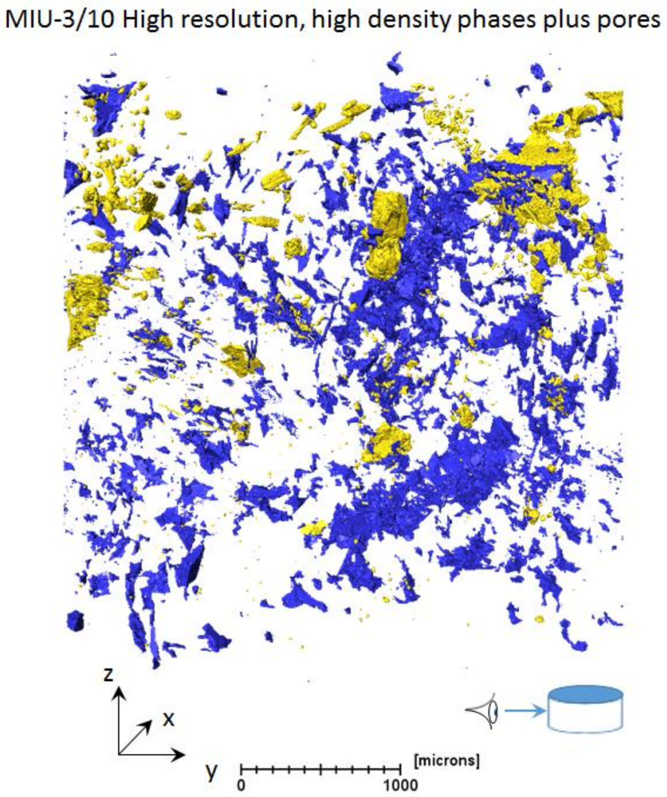
MIU-3/10 High resolution, high density phases plus pores
z
x
y
0
1000
[microns]

P.

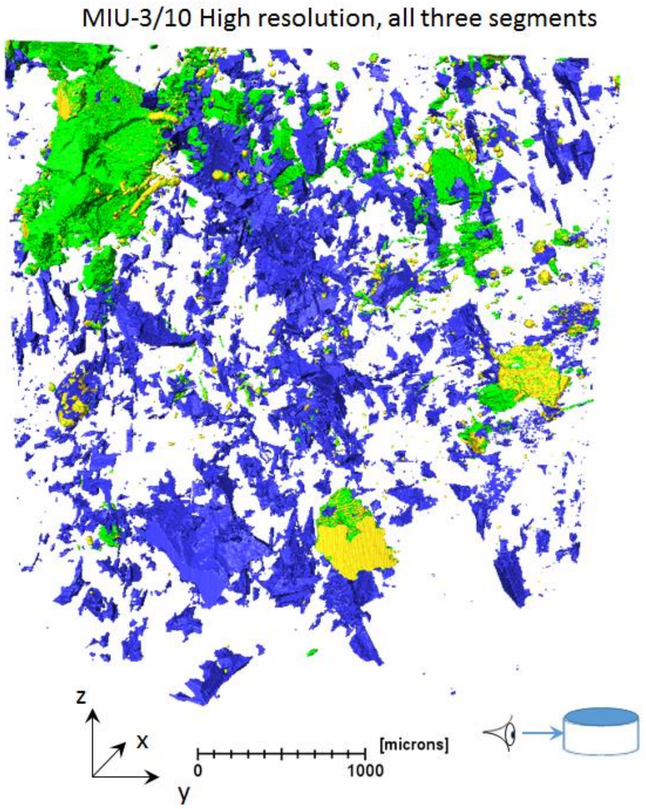
MIU-3/10 High resolution, all three segments
z
x
y
0
1000
[microns]

Q.

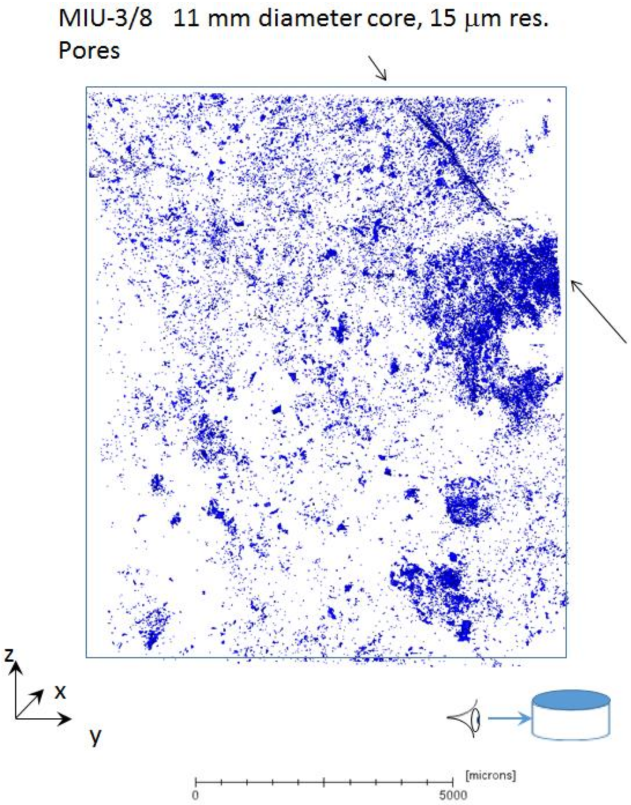
MIU-3/8 11 mm diameter core, 15 μm res.
Pores
z
x
y
0
5000
[microns]

R.

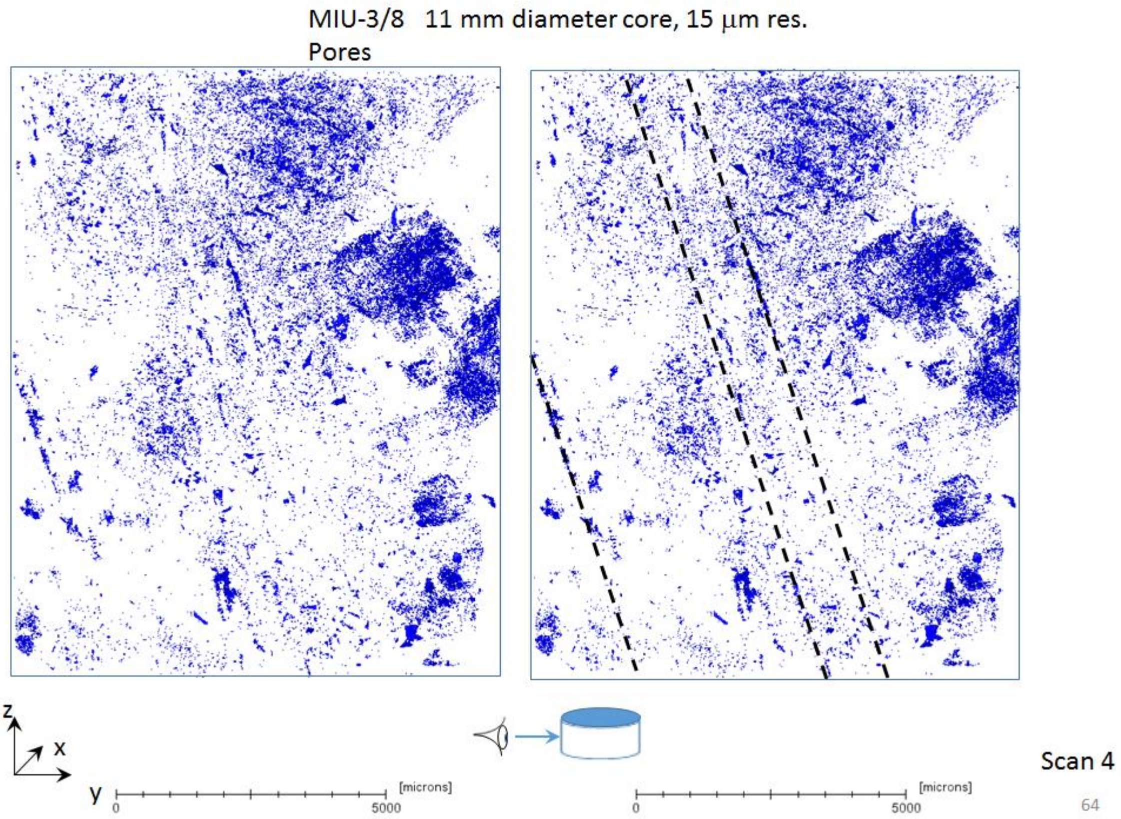
MIU-3/8 11 mm diameter core, 15 μm res.
Pores
z
x
y
0
5000
[microns]
0
5000
[microns]
Scan 4
64

S.

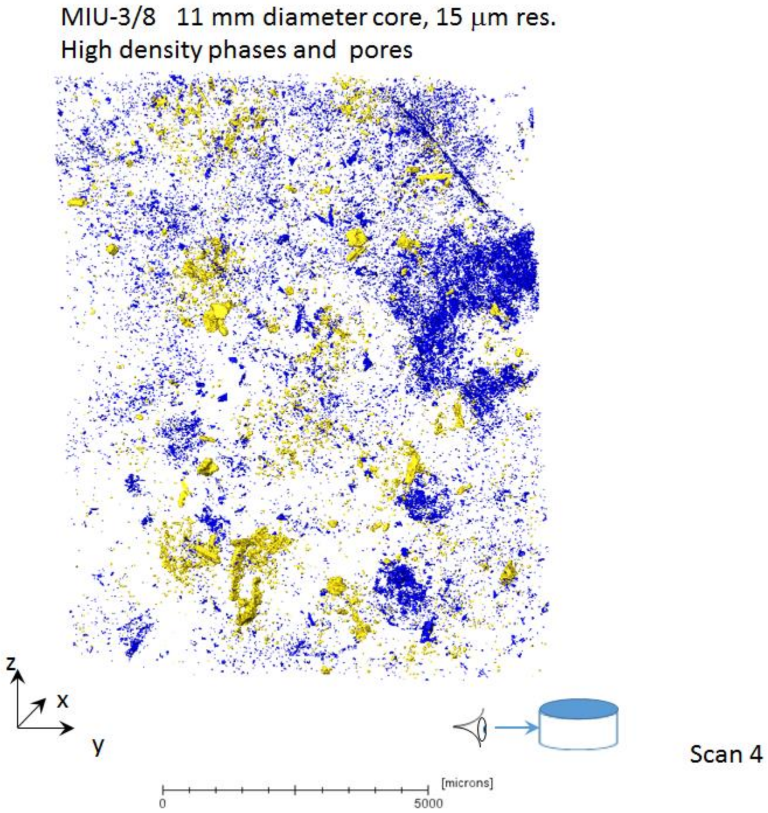
MIU-3/8 11 mm diameter core, 15 μm res.
High density phases and pores
z
x
y
Scan 4
[microns]
0
5000

T.

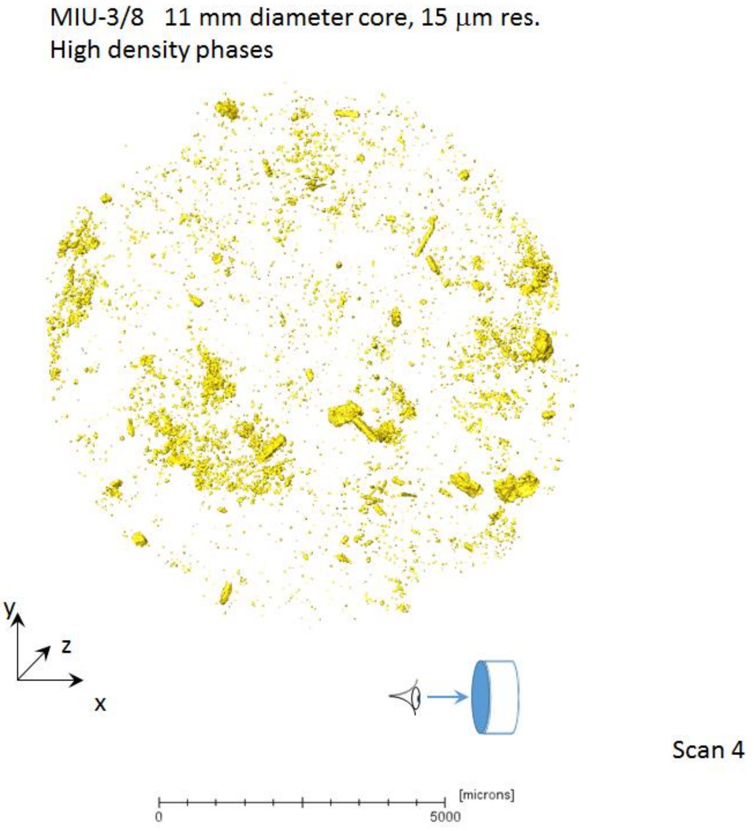
MIU-3/8 11 mm diameter core, 15 μm res.
High density phases
y
z
x
Scan 4
[microns]
0
5000

U.

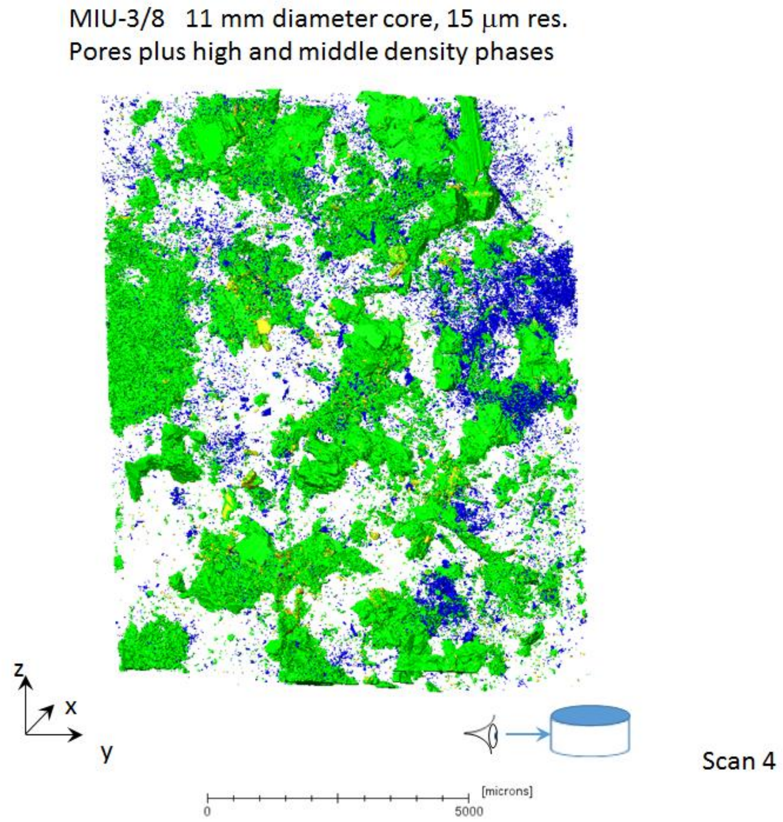
MIU-3/8 11 mm diameter core, 15 μm res.
Pores plus high and middle density phases
z
x
y
Scan 4
[microns]
0
5000

V.

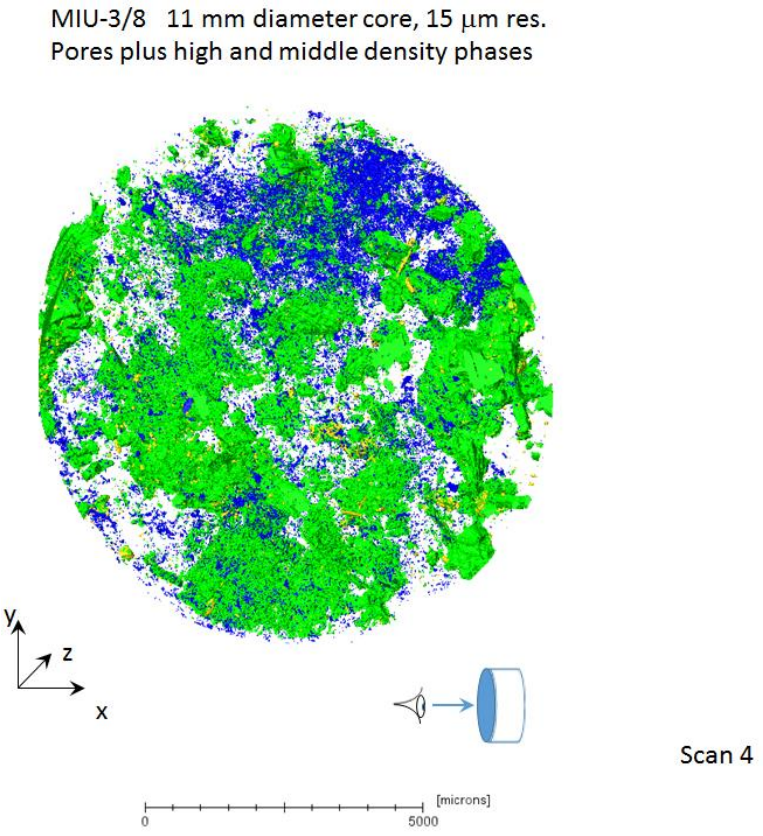
MIU-3/8 11 mm diameter core, 15 μm res.
Pores plus high and middle density phases
y
z
x
Scan 4
[microns]
0
5000

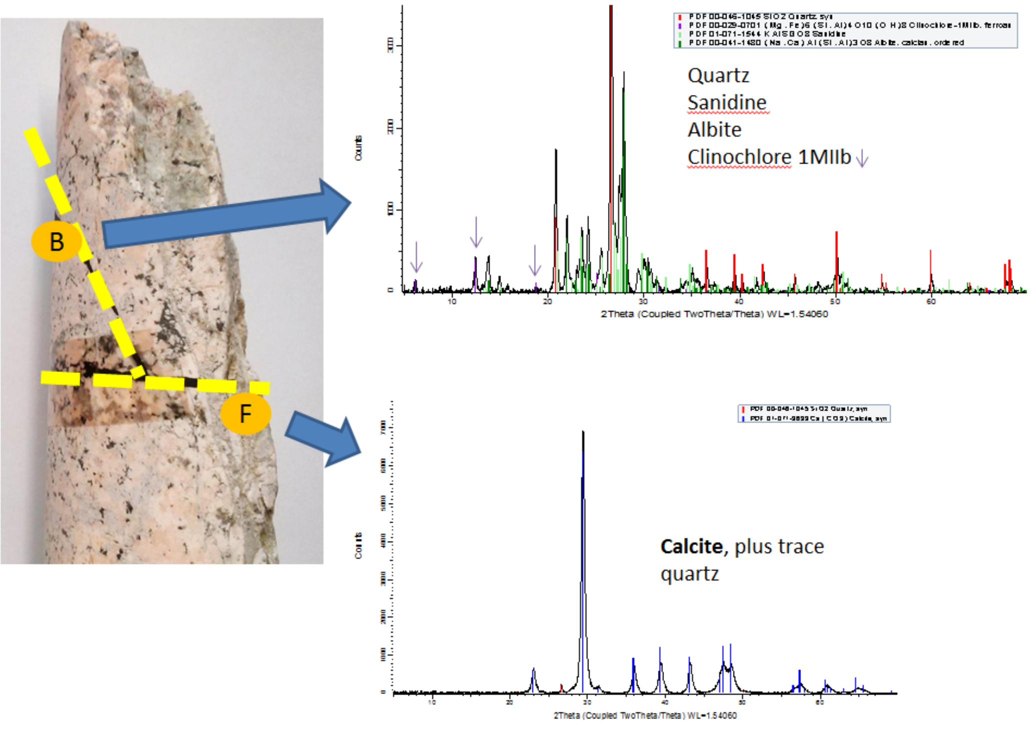

Supplementary Figure 2. XRD identifies calcite as the most important phase volumetrically within the well-developed fracture infill. Approximately 3 cm away from the fracture the dominant secondary phase determined by XRD is clinochlore. Sericite is the only other secondary phase identified by XRD in MIU-3/10. Upper XRD profile is for the bulk (B), lower is for the fracture infill (F).

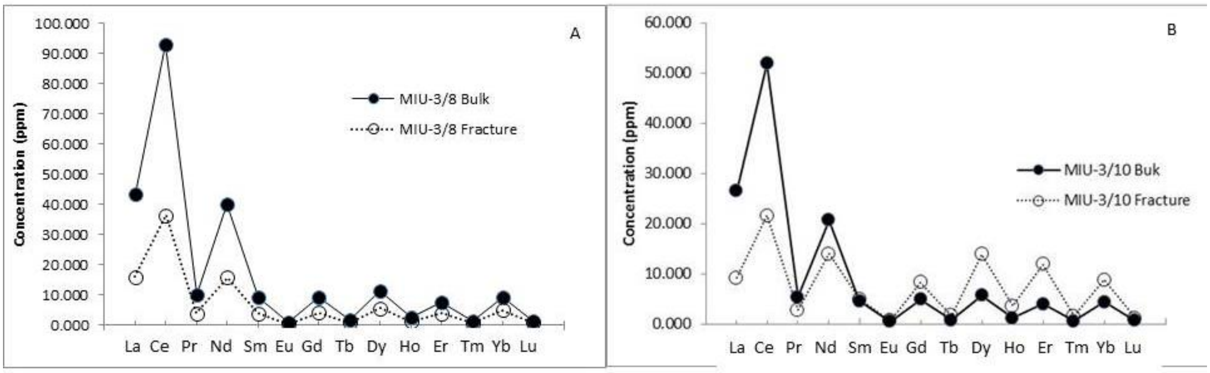

Supplementary Figure 3. A) REE patterns measured by ICP-MS for MIU-3/8 bulk (solid symbols) and fracture infill (hollow symbols) The fracture infill has lower concentrations of all REE. B) REE patterns for MIU-3/10 bulk (solid symbols) and fracture infill (hollow symbols) The fracture has higher HREE/LREE compared to the bulk.

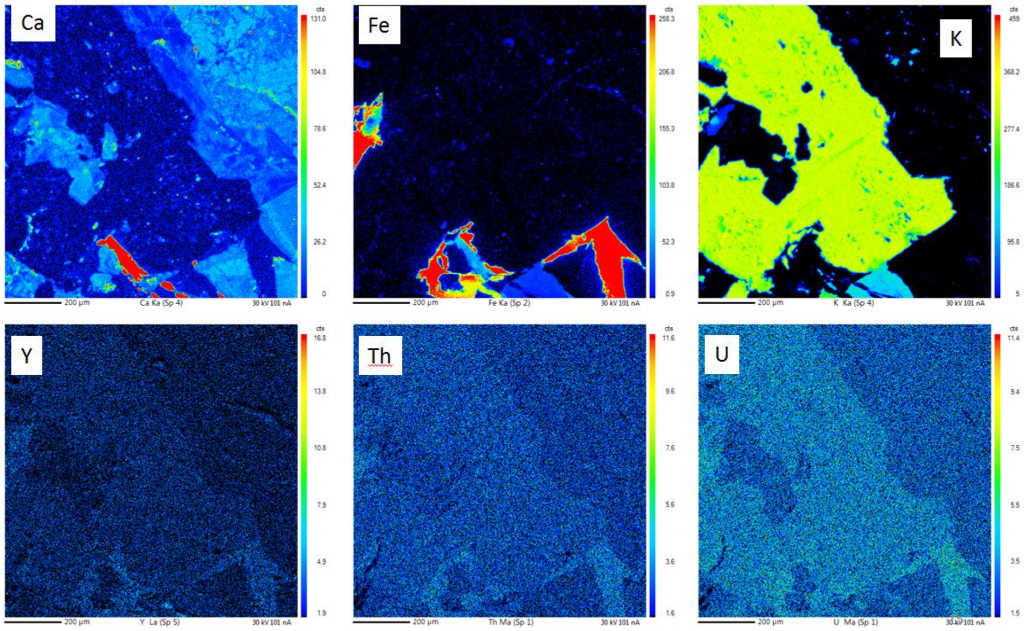

Supplementary Figure 4. EMPA map of MIU-3/10 taken 20 mm away from main fracture. Here, the primary phases are relatively pristine and there is no evidence of pervasive alteration.

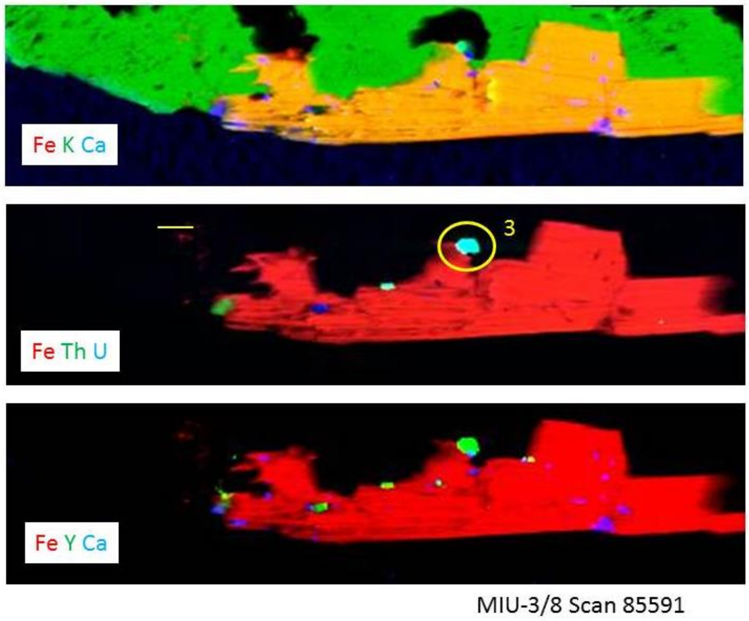

Supplementary Figure 5. MIU-3/8, microfocus XRF map area 2, pristine. False colour elemental map, with colour key provided as element symbols coloured to correspond to map colours. In the top image, high Fe and K biotite appears orange, K-feldspar is green, with small accessory Ca-rich phase in blue (most probably a carbonate or phosphate mineral). The circled grain labelled 3 is rich in both Th and U and is the third actinide oxide point analysis given in Table 2 above. 100 micron scale bar.

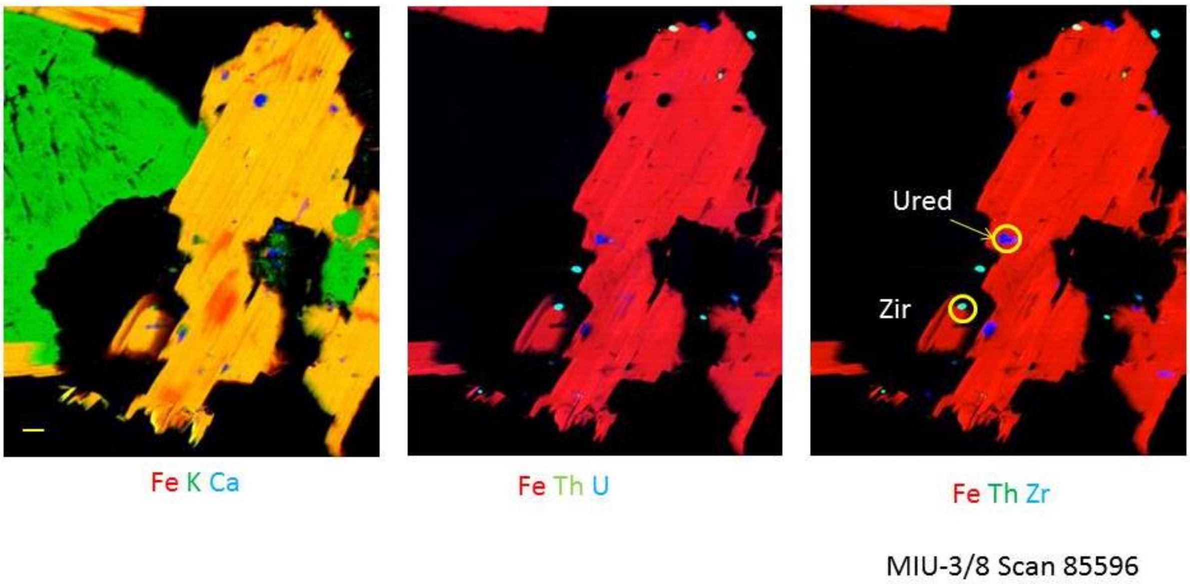

Supplementary Figure 6. MIU-3/8 microfocus XRF map area 3, pristine. False colour elemental map, with colour key provided as element symbols coloured to correspond to map colours. Major element map at left identifies biotite as the orange coloured phase and K-feldspar as the green minerals. The central panel shows a number of hotspots for U and Th. Right hand panel shows that U and Th correlate with Zr and therefore hosted within zircons in this area. XANES spectrum location for reduced U is labelled as ‘Ured’, as is the Table 2 zircon point analysis location (Zir). 100 micron scale bar.

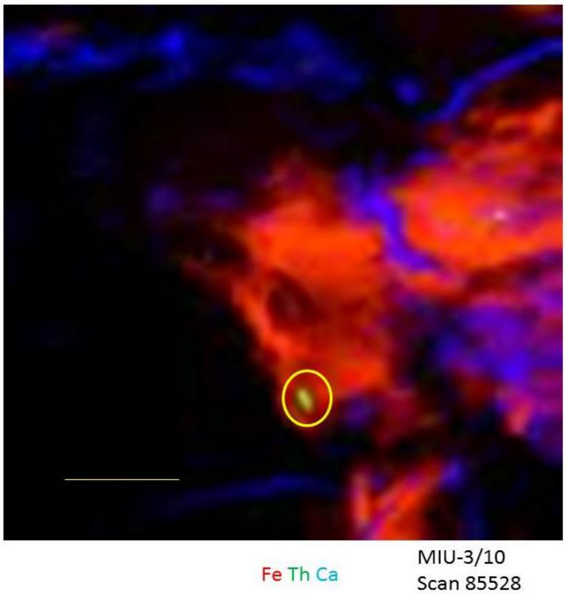

Supplementary Figure 7. MIU-3/10 microfocus XRF map area 2. False colour elemental map, with colour key provided as element symbols coloured to correspond to map colours. Again, blue shows the sinuous calcite infill within the fractures and a small Th-rich grain (circled). 100 micron scale bar

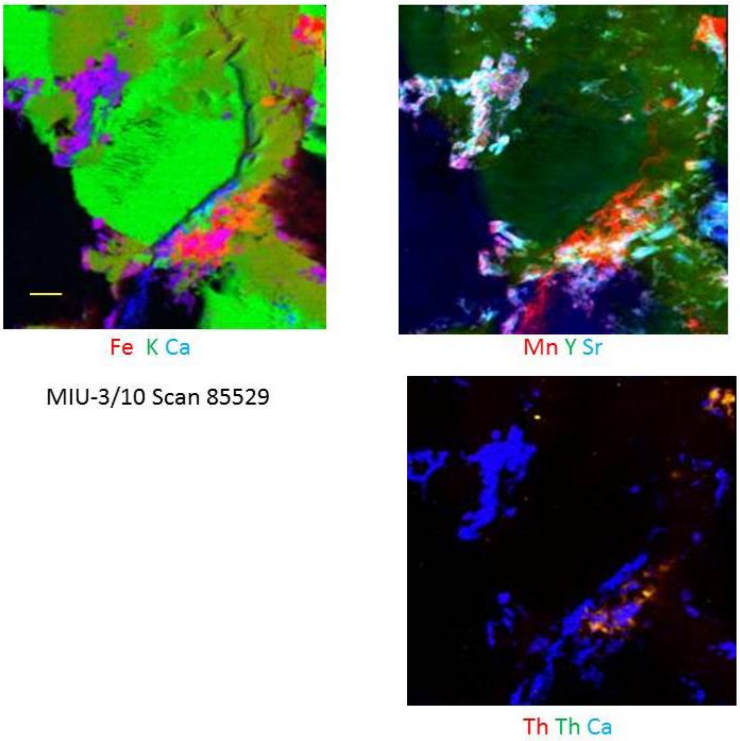

Supplementary Figure 8. MIU-3/10 microfocus map area 3. False colour elemental map, with colour key provided as element symbols coloured to correspond to map colours. Upper left major element map shows K-feldspar in green, iron oxyhydroxide in red, and calcite in the fractures as blue. The upper right panel again shows that Mn correlates with Fe-oxyhydroxides (orange coloured areas), but also shows that calcite precipitates may be rich in Mn, Y, and Sr (white areas). As in the other areas, hotspots of Th are again associated with carbonate (lower right panel). 100 micron scale bar.

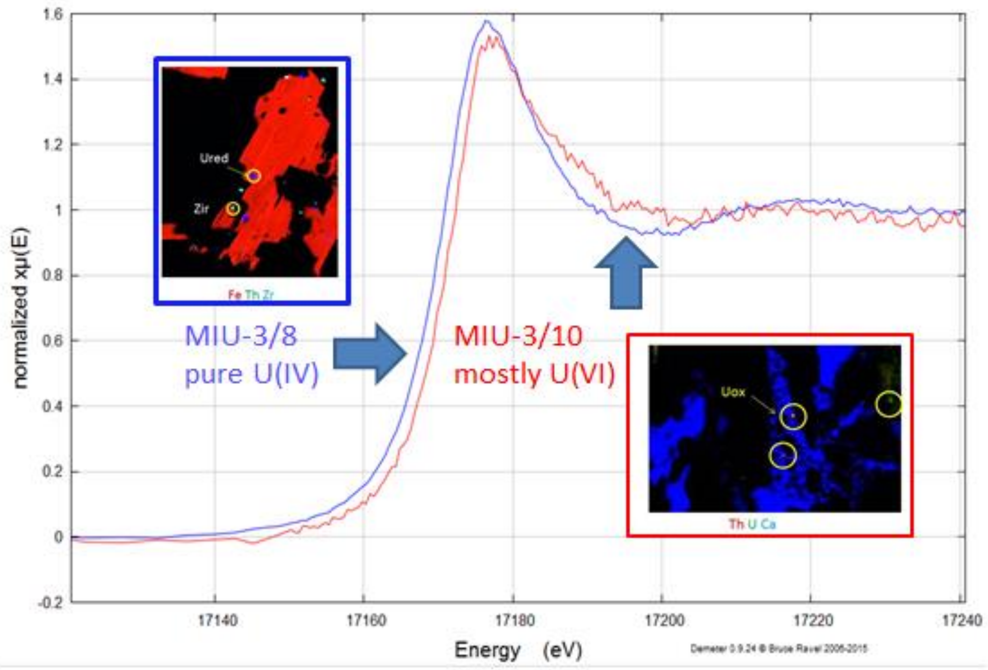

Supplementary Figure 9. U L-III XANES spectra. The blue curve is for uranium located within specimen MIU-3/8, near the main fracture surface but taken from a small uranium and zirconium-rich grain associated with a large biotite crystal. Uranium correlates with Zr in this region and we therefore conclude this is from a primary zircon crystal with appreciable U substitution. Figure 5

(scan 85596) shows the region mapped and the U-Zr association, point analysis is presented on Table 2. The red curve is from specimen MIU-3/10, a sample that has undergone significantly more hydrous alteration than MIU-3/8. Here, the spectrum was obtained from within an altered vein proximate to the main fracture that was relatively rich in U. (A detailed map of this area is provided as Figure 6-scan 85508: the U spectrum originates from the small U-rich grains in the center of the map associated with the calcite infill).

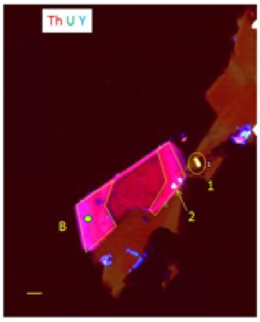

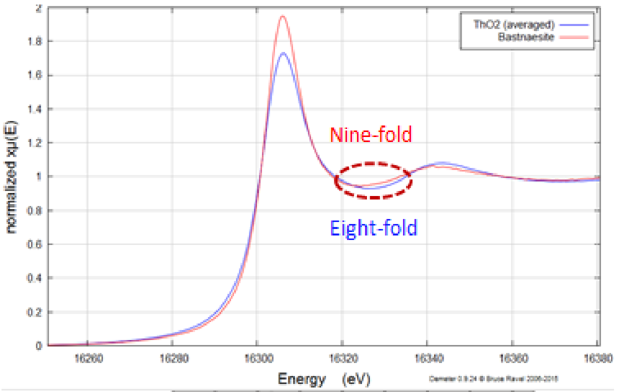

Blue curve is from a primary ThO_2 grain within MIU-3/8 labelled "1". In contrast, the red curve is from bastnaesite, point labelled "B".

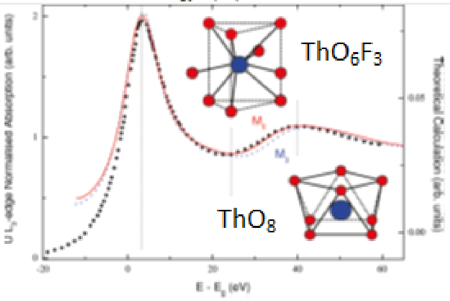

Lower panel is Figure 4 from Chaboy and Diaz-Moreno (2011). It shows the subtle differences in spectra expected between a tetravalent 9-fold (M6) and 8-fold (M3) coordinated actinide. Our XANES data resolve this difference between bastnaesite and thorium oxide.

Supplementary Figure 10. Th XANES. Thorium XANES were obtained for primary bastnaesite and thorium oxide within MIU-3/8 (Figure 5A, corresponding to Table S6 AcO_2 point analyses 1 and 2). Here subtle differences between the data and theoretical calculated spectra for actinides[51] indicate that primary Th is substituting into the 9-fold Ce site in bastnaesite [CeO_6F_3], while it is in the expected 8-fold coordination environment within the actinide oxides [ThO_8].

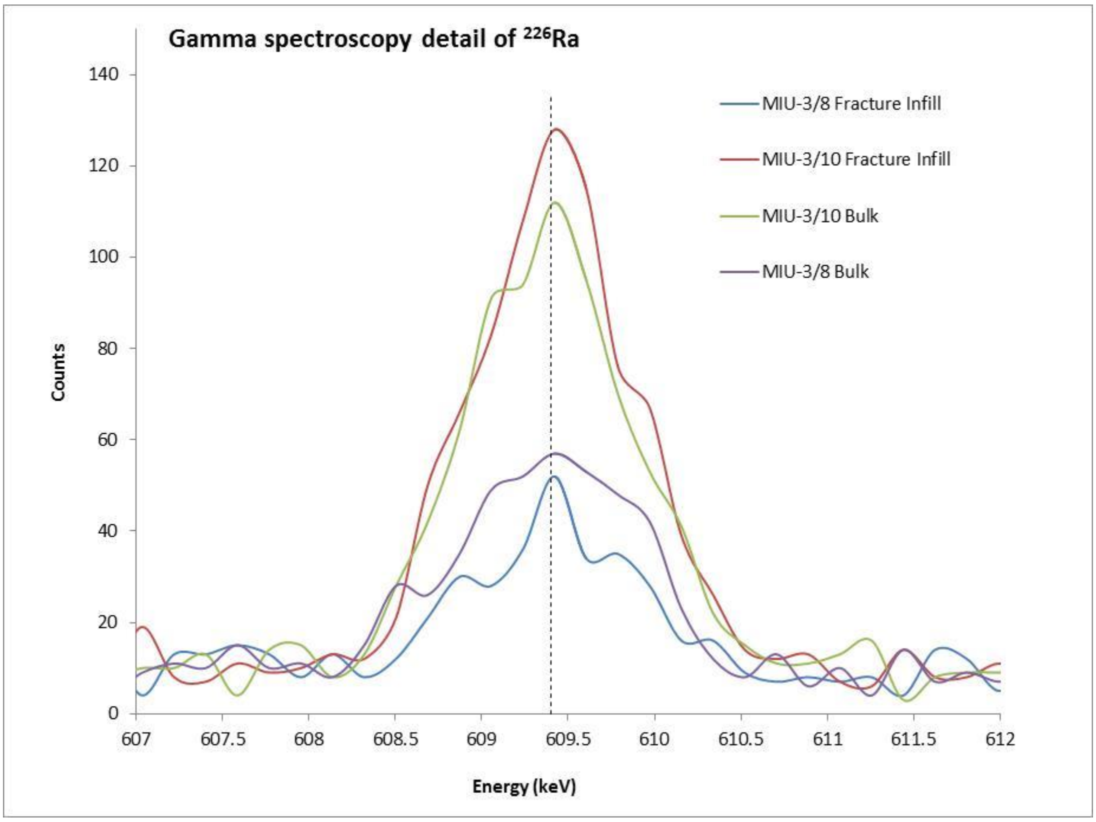

Supplementary Figure 11. Gamma spectrometry
